# Indirubin attenuates sepsis by targeting the EGFR/SRC/PI3K and NF-κB/MAPK signaling pathways in macrophages

**DOI:** 10.3389/fphar.2025.1542061

**Published:** 2025-03-12

**Authors:** Yancen Li, Chengyu Wan, Fan Li, Guang Xin, Tao Wang, Qilong Zhou, Tingyu Wen, Shiyi Li, Xiaoting Chen, Wen Huang

**Affiliations:** ^1^ West China Center of Excellence for Pancreatitis, Institute of Integrated Traditional Chinese and Western Medicine, Natural and Biomimetic Medicine Research Center, Tissue-Orientated Property of Chinese Medicine Key Laboratory of Sichuan Province, West China School of Medicine, West China Hospital, Sichuan University, Chengdu, China; ^2^ Animal Experimental Center, West China Hospital, Sichuan University, Chengdu, China

**Keywords:** *Isatidis Folium*, indirubin, sepsis, inflammation, network pharmacology

## Abstract

**Background:**

*Isatidis Folium*, a botanical drug widely used in traditional medicine, is known for its anti-inflammatory properties, including heat-clearing, detoxifying, and blood-cooling effects. Although its potential in sepsis treatment has been suggested, the bioactive metabolites and underlying mechanisms remain poorly understood.

**Methods:**

Network pharmacology and molecular docking were employed to identify the therapeutic effects and mechanisms of Indirubin, the major bioactive metabolite of *Isatidis Folium*, in sepsis treatment. *In vivo*, a cecal ligation and puncture (CLP)-induced mouse sepsis model was used to evaluate the protective effects of Indirubin through histopathological analysis, ELISA, and biochemical assays. *In vitro*, RAW264.7 cells were stimulated with LPS and treated with varying concentrations of Indirubin. The anti-inflammatory effects of Indirubin were assessed using ELISA, apoptosis assays, and Western blotting.

**Results:**

Network pharmacology analysis identified Indirubin as the major bioactive metabolite of *Isatidis Folium* and EGFR and SRC as its key molecular targets. Experimental validation demonstrated that Indirubin significantly improved survival rates, alleviated tissue injury, and suppressed inflammatory responses in sepsis models. Mechanistically, Indirubin inhibited LPS-induced activation of the EGFR/SRC/PI3K and NF-κB/MAPK pathways in macrophages, significantly reducing cell death and inflammation in RAW264.7 cells.

**Conclusion:**

Indirubin, the primary bioactive metabolite of *Isatidis Folium*, exerts protective effects against sepsis by targeting the EGFR/SRC/PI3K and NF-κB/MAPK signaling pathways in macrophages. These findings provide a mechanistic basis for the development of Indirubin as a multi-target therapeutic agent for sepsis treatment.

## Graphical Abstract

**Figure:**
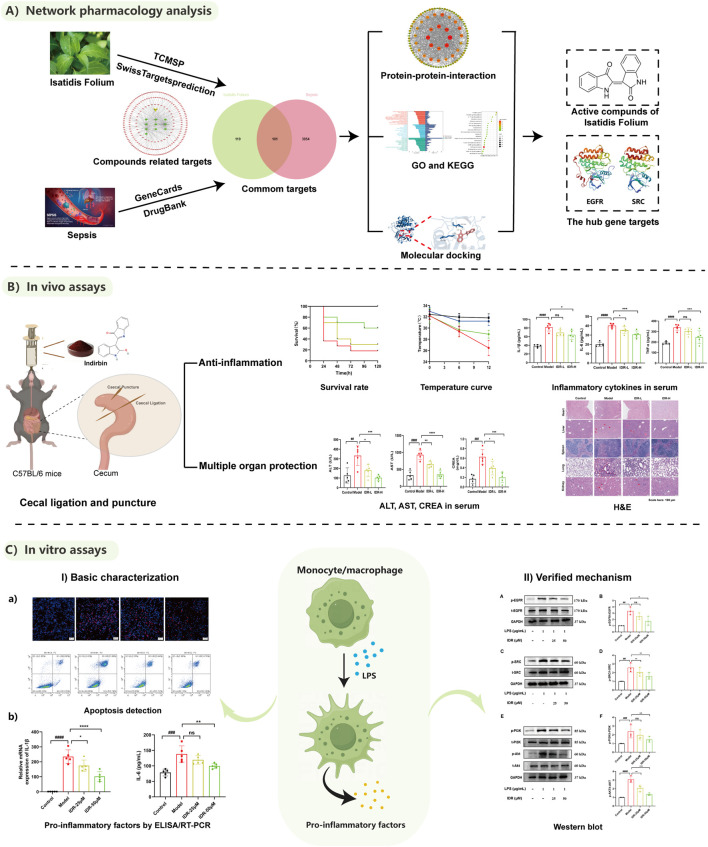
Screening and mechanism exploration of main components in the treatment of sepsis by *Isatidis Folium*.

## 1 Introduction

Sepsis is a severe and life-threatening syndrome that affects more than 19 million people worldwide, accounting for more than six million deaths, with mortality rates exceeding 25% (Pei et al., 2022). It is characterized by dysregulation of the immune response to infection, triggering excessive inflammatory reactions and complications that often progress to multiple organ dysfunction syndrome (MODS) (Srdić et al., 2024; van der Poll et al., 2017). The persistent and poorly controlled inflammatory response is a major driver of complications, disease progression, and high mortality rates in sepsis. Consequently, there is an urgent need for novel, reliable, and effective anti-inflammatory therapies to improve the management and outcomes of sepsis.

*Isatidis Folium* [Isatis tinctoria subsp. tinctoria (Brassicaceae)], the dried leaf of the cruciferous plant *Isatis tinctoria L.* (Brassicaceae), is a botanical drug traditionally used to purge heat, detoxify, cool blood, and alleviate skin conditions (Hsieh et al., 2021). Clinically, it is widely employed in the treatment of parotitis, sore throat (Yao et al., 2020), erysipelas, numbness and rash (Chen et al., 2022a; Deng et al., 2013; Xu et al., 2004). Modern pharmacological studies have identified its primary metabolites as alkaloids (Liu et al., 2018; Chen et al., 2012), organic acids (Wu et al., 1997), and flavonoids (Deng et al., 2008), which exhibit diverse biological activities, including antibacterial, anticancer, antiaging, antioxidant, anti-inflammatory, and immunomodulatory effects (Dong et al., 2015). Evidence suggests that aqueous extracts of *Isatidis Folium* ameliorate skin inflammation in models resembling atopic dermatitis by down-regulating pro-inflammatory cytokines and chemokines (Min et al., 2023). Additionally, the n-butanol fraction of *Isatidis Folium* has been shown to significantly inhibit LPS-induced inflammatory cytokine production in macrophages and protect against LPS-induced endotoxic shock in mice (Dong et al., 2015). These findings indicate that the anti-inflammatory metabolites in *Isatidis Folium* may serve as a promising source for sepsis therapeutics. However, the specific active metabolites and their underlying mechanisms remain poorly understood. This study aims to identify potential active metabolites in *Isatidis Folium* through integrated screening and experimental validation, focusing on their therapeutic effects and mechanisms against sepsis.

Network pharmacology is an interdisciplinary approach that integrates principles from pharmacology and systems biology, focusing on the molecular interactions between bioactive metabolites, proteins, genes, and diseases. This method aligns well with the study of traditional Chinese medicine (TCM), which often involves multiple metabolites and targets, by providing a comprehensive analytical framework to elucidate the molecular mechanisms underlying TCM’s therapeutic effects (Wang et al., 2024; Liu et al., 2024a; Feng et al., 2023). It has emerged as a critical strategy in TCM research (Qiu et al., 2024; Jiang et al., 2024). To validate the reliability of targets identified through network pharmacology, molecular docking serves as a key methodological tool (Zhang et al., 2021). The integration of network pharmacology and molecular docking offers a robust approach to uncovering the therapeutic principles of TCM.

In this study, we focused on *Isatidis Folium*, employing network pharmacology and molecular docking to screen its bioactive metabolites and conduct a comprehensive analysis of potential targets and disease-related pathways associated with sepsis. Using GO functional enrichment analysis, KEGG pathway enrichment analysis, and protein-protein interaction (PPI) analysis, we constructed a “metabolite-target-pathway” network to predict the therapeutic effects and mechanisms of *Isatidis Folium* and its bioactive metabolites on sepsis. Based on the screening results, we selected the top-ranked metabolite, Indirubin, for experimental validation. *In vitro* experiments demonstrated that Indirubin significantly reduced LPS-induced cell death. *In vivo* studies confirmed that Indirubin markedly decreased the mortality rate of septic mice induced by cecal ligation and puncture (CLP), reduced serum levels of inflammatory factors, and alleviated organ damage in septic mice. These findings provide novel insights into the development of more effective therapeutic strategies for sepsis.

## 2 Materials and methods

### 2.1 Reagents and antibodies

Reagents used for this study were purchased commercially: Indirubin (IDR, I111268) was purchased from Shanghai Aladdin Biochemical Technology Co., Ltd. (Shanghai, China). Cell counting kit-8 (CCK-8, C0037), Erlotinib (SC0168) and Dexamethasone (DXMS, ST1254) were obtained from Beyotime Biotechnology (Shanghai, China). The EGFR tyrosine kinase inhibitor PD168393 (PD, PZ0285) was supplied by Sigma-Aldrich (St Louis, MO, United States). The Annexin V-FITC/PI Apoptosis Kit (E-CK-A211) was purchased from Elabscience Biotechnology Co., Ltd. (Wuhan, China). Hoechest 33342 and PI were bought from Molecular Probes (Eugene, Oregon, United States). Protease inhibitor cocktail tablets (118361700001) were from Roche Diagnostics (Mannheim, Germany). The amounts of inflammatory factors (IL-6, IL-1β and TNF-α) were quantified utilizing ELISA kits provided by Thermo Fisher Scientific, MD, United States. Antibodies used for WB are commercially available (Supplementary Table S1).

### 2.2 Data collection

Using the TCMSP (Traditional Chinese Medicine System Pharmacology) dataset and analytic platform (https://old.tcmsp-e.com/tcmsp.php), we searched for “*Isatidis Folium*” to retrieve information on its bioactive metabolites. The selection criteria for these metabolites included oral bioavailability (OB) ≥ 30% and drug similarity (DL) ≥ 0.18. The resulting metabolites were further supplemented with additional compounds identified in prior studies, as detailed in Supplementary Table S2. Targets associated with each metabolite were predicted using the Swiss Target Prediction database and standardized using the UniProtKB database using the keyword “sepsis” and similarly standardized using UniProtKB database. Common targets between *Isatidis Folium* metabolites and sepsis were identified using Venny 2.1.0. Finally, the data were imported into Cytoscape (version 3.9.1) to construct a network illustrating the interactions among bioactive metabolites, targets, and diseases.

### 2.3 PPI network construction

Core targets were identified through protein-protein interaction (PPI) network analysis. The STRING platform was utilized with the highest confidence level set as 0.900 and the species specified as “*Homo sapiens*”. The intersection targets were uploaded to STRING to generate a PPI network, which was then imported into Cytoscape (version 3.9.1) for further analysis. Using the Maximal Clique Centrality (MCC) algorithm within the CytoHubba plugin, the top ten genes with the highest degree values were selected as core genes.

### 2.4 Molecular docking

Molecular docking was performed between putative bioactive metabolites (Indirubin, poriferast-5-en-3beta-ol, 6-(3-oxoindolin-2-ylidene)indolo [2,1-b]quinazolin-12-one, beta-sitosterol and Glycyrol) and key targets (CASP3, EGFR, SRC, HSP90AA1 and ESR1). The protein structures of the core targets were obtained from the PDB database (RCSB PDB: Homepage), with the following PDB IDs: CASP3 (2y0b), EGFR (8sc7), SRC (2bdj), HSP90AA1 (1byq), and ESR1 (5acc). Using Pymol (version 2.3.0), ligands and water molecules were removed from the target proteins. The protein structures were then imported into AutoDockTools (version 1.5.6) for preprocessing, including hydrogenation, charge distribution calculation, and atomic type assignment. Molecular docking results were visualized using Pymol. The binding affinity between proteins and ligands was quantified by the binding energy of the docked complexes.

### 2.5 Cells

RAW264.7 murine macrophages were obtained from the American Type Culture Collection (ATCC) and maintained in Dulbecco’s Modified Eagle’s Medium (DMEM) supplemented with 10% fetal bovine serum (FBS) at 37°C in a humidified atmosphere with 5% CO_2_. Cells were plated at an appropriate density and allowed to adhere overnight before experimental manipulation. For the experiments, cells were divided into the following groups: control, model (LPS, 1 μg/mL), IDR (25 μM and 50 μM), Dexamethasone (10 μM), PD168393 (10 μM),and PD168393 + IDR (PD168393, 10 μM; IDR, Indirubin, 50 μM).

(1) Model group: Cells were treated with lipopolysaccharide (LPS) at a concentration of 1 μg/mL for 24 h to induce an inflammatory response.
(2) IDR group (Indirubin, 25 μM and 50 μM): Cells were treated with 25 μM or 50 μM Indirubin and 1 μg/mL LPS for 24 h.
(3) Dexamethasone group (10 μM): Cells were treated with 10 μM Dexamethasone and 1 μg/mL LPS for 24 h.
(4) PD168393 group (10 μM): Cells were pretreated with EGFR inhibitor PD168393 (10 μM) for 6 h, after which the cells were treated with LPS (1 μg/mL) for 24 h in the presence of the inhibitor.
(5) PD168393 + IDR group (PD168393, 10 μM; IDR, Indirubin, 50 μM): Cells were pretreated with EGFR inhibitor PD168393 (10 μM) for 6 h, after which the cells were treated with LPS (1 μg/mL) and Indirubin (50 μM) for 24 h in the presence of the inhibitor.

### 2.6 Animals

Male C57BL/6 specific-pathogen-free (SPF) mice (8 weeks old, 23 ± 2 g) were purchased from Zhejiang Vital River Laboratory Animal Technology Co., Ltd. (Animal License No. SCXK Zhe 2019–0001). The mice were housed in the Animal Laboratory Center of West China Hospital under controlled environmental conditions: temperature 22.5°C ± 2.5°C, humidity 50% ± 5%, and a 12-h light/dark cycle. Food and water were provided *ad libitum*. All experimental procedures were approved by the Ethics Committee of West China Hospital, Sichuan University (Approval No. 20240704007) and conducted in accordance with the National Institutes of Health Guide for the Care and Use of Laboratory Animals.

### 2.7 Cecal ligation and puncture

Sepsis mouse models were established via cecal ligation and puncture (CLP) according to established protocols (Kiwit et al., 2024; Yu et al., 2022; Lv et al., 2021; Rittirsch et al., 2009). Mice were randomly divided into the following groups: control, model, IDR-L (Indirubin low dose, 10 mg/kg), IDR-H (Indirubin high dose, 20 mg/kg), Dexamethasone (DXMS, 10 mg/kg), Erlotinib (45 mg/kg), and Erlotinib + Indirubin (Erlotinib, 45 mg/kg; Indirubin 20 mg/kg).

(1) Indirubin groups (IDR-L: 10 mg/kg and IDR-H:20 mg/kg): mice received intragastric administration of the respective doses for three consecutive days prior to modeling, followed by an additional dose post-modeling.
(2) Dexamethasone group (10 mg/kg): mice were given intraperitoneal injection 1.5 h after molding.
(3) Erlotinib group (45 mg/kg): mice received intragastric administration for three consecutive days prior to modeling, followed by an additional dose post-modeling.
(4) Erlotinib + Indirubin group (Erlotinib, 45 mg/kg; Indirubin 20 mg/kg): mice received intragastric administration of the respective agents for three consecutive days prior to modeling, with Erlotinib being administered intragastrically 2 h before Indirubin, followed by an additional dose post-modeling.

The mice were fasted for 12 h before the procedure. Anesthesia was induced using a 1% pentobarbital solution. The abdominal cavity was surgically opened, and the distal cecum was ligated and punctured. A fecal sample was collected to confirm patency, after which the cecum was returned to the abdominal cavity, and the incision was sutured. After the procedure, the mice were injected subcutaneously with 0.9% sodium chloride solution (1 mL/20 g) into the abdominal cavity, resuscitation was performed with a heat blanket until the mice regained consciousness, and then placed back in the cage. In the sham group, mice underwent laparotomy and suturing without cecal ligation or puncture.

### 2.8 Murine sepsis score (MSS) of mice

Based on the MSS scoring system (Liu et al., 2024b; Sulzbacher et al., 2022) (Supplementary Table S3), as the score increases, the condition of the mice becomes more severe. The MSS system evaluates seven factors: response to stimulus, eyes, activity, respiratory quality, respiratory rate, level of consciousness, and appearance (Kathem et al., 2024). This grading system is a great tool for evaluating the welfare of mice and determining their overall health. Each scientist individually assessed every experimental mouse, and the severity of the mice’s condition was determined by calculating the average score.

### 2.9 Measurement of lung wet/dry ratio

The wet weight of the left lung was quantified. The sample is then dehydrated in a 60°C oven for an interval of 48 h until it reaches the intended level of moisture removal. Pulmonary edema can be determined by dividing the dry weight of the lung by its wet weight to obtain the lung water content (Zhou et al., 2018).

### 2.10 Enzyme-linked immunosorbent assay (ELISA)

The quantification was performed according to the directions provided by the manufacturer. After allowing the kit to equilibrate at ambient temperature for at least half an hour, dilute the concentrated washing solution with distilled water. The samples were introduced into the plate that contained the enzyme for the purpose of initiating a reaction. Following the washing process, the antibodies labeled with enzymes were introduced and allowed to incubate at a temperature of 37°C for a duration of 30 min. Following the washing process, the color developer A and B were thoroughly mixed and then incubated at an environment of 37°C for 15 min to facilitate color development. Ultimately, the enzyme-labeled instrument was utilized to measure the absorbance value at a wavelength of 450 nm.

### 2.11 Hematoxylin and eosin (H&E) staining

The lungs, livers and kidneys of mice were fully soaked in a 4% paraformaldehyde solution. After a duration of 24 h, apply hematoxylin dye for a period of 3 min, followed by eosin dye for a period of 2 min. Ultimately, the discolored tissue was meticulously analyzed utilizing an optical microscope produced by an upright fluorescence microscope (Axio Imager Z2, Zeiss, Oberkochen, Germany).

### 2.12 Real-time polymerase chain reaction (RT-PCR)

The total amount of RNA was dissociated using TRIzol (Thermo Fisher Scientific, MA, United States) and then purified using an RNA purification kit (Thermo Fisher Scientific, MA, United States). Subsequently, complementary DNA (cDNA) was generated utilizing the procedures outlined in prior research (Otten et al., 2021). The target mRNAs were identified using the SYBR^®^ Green I master mix in an RT-PCR analysis equipment (ABI QuantStudio 7, Thermo Fisher, United States). The expression levels of all target mRNAs were compared to the expression levels of β-actin, which served as the control (Table 1).

**TABLE 1:** PCR primer sequences.

Gene symbol	Accession number	Forward primer (5′–3′)	Reverse primer (5′–3′)	Expected amplicon size (base pair)
β-actin	NM_007393	GTG​CTA​TGT​TGC​TCT​AGA​CTT​CG	ATG​CCA​CAG​GAT​TCC​ATA​CC	174
IL-6	NM_031168.2	AGT​TCC​TCT​CTG​CAA​GAG​ACT​TCC	TTG​CCA​TTG​CAC​AAC​TCT​TTT​C	214
IL-1β	NM_008361.4	TGC​CAC​CTT​TTG​ACA​GTG​ATG	AAG​GTC​CAC​GGG​AAA​GAC​AC	220
TNF-α	NM_013693.3	TCA​AAA​TTC​GAG​TGA​CAA​GCC​TG	GGT​ATG​AGA​TAG​CAA​ATC​GGC​TG	245

### 2.13 Flow cytometry

The apoptosis detection tool utilized was the Annexin V-FITC/propidium iodide (PI) cell apoptosis detection kit supplied from Elabscience, China. The cells of different groups were suspended in 195 μL solution and subsequently labeled with 5 μL of Annexin V-fluorescein isothiocyanate isomer (FITC) and PI staining solution. The procedure was conducted for a duration of 10 min at ambient temperature in the absence of illumination.

### 2.14 Western blot analysis

Cellular protein samples were extracted using RIPA lysis buffer, and protein quantification was performed using the bicinchoninic acid (BCA) assay (Beyotime Biotechnology). Equal amounts of ptotein from each sample were separated by SDS-PAGE and transferred onto a polyvinylidene fluoride (PVDF) membrane (Millipore, Bedford, MA, United States). The membrane was blocked at room temperature for 1 h and then incubated with the primary antibody at 4°C overnight. After washing three times, the membrane was incubated with the second antibody at room temperature for 1 h. Protein bands were visualized and analyzed using the Image Lab software (Bio-Rad Laboratories, United States).

### 2.15 Statistical analysis

The data was processed using Graphpad Prism 9.0 and presented as mean value plus or minus the standard deviation. The Shapiro-Wilk test was employed to ascertain normality, while an unpaired Student’s t-test with Welch’s correction was utilized to evaluate statistical significance between two groups if normality was confirmed and no significant variance differences were found. Additionally, to evaluate the statistical significance among multiple groups, we utilized a One-way ANOVA with Dunnet’s post-test analysis. An ANOVA was performed in two ways, and Sidak’s multiple comparison correction was used if needed. The data was analyzed using a two-tailed Mann-Whitney U-test or a Kruskal Wallis test with Dunn’s multiple comparison correction if it did not follow a normal distribution. This research did not include any randomization or blinding techniques. Statistically noteworthy variations were established by applying a p-value threshold of l 0.05 or lower.

## 3 Results

### 3.1 Screening of potential targets

**FIGURE 1:**
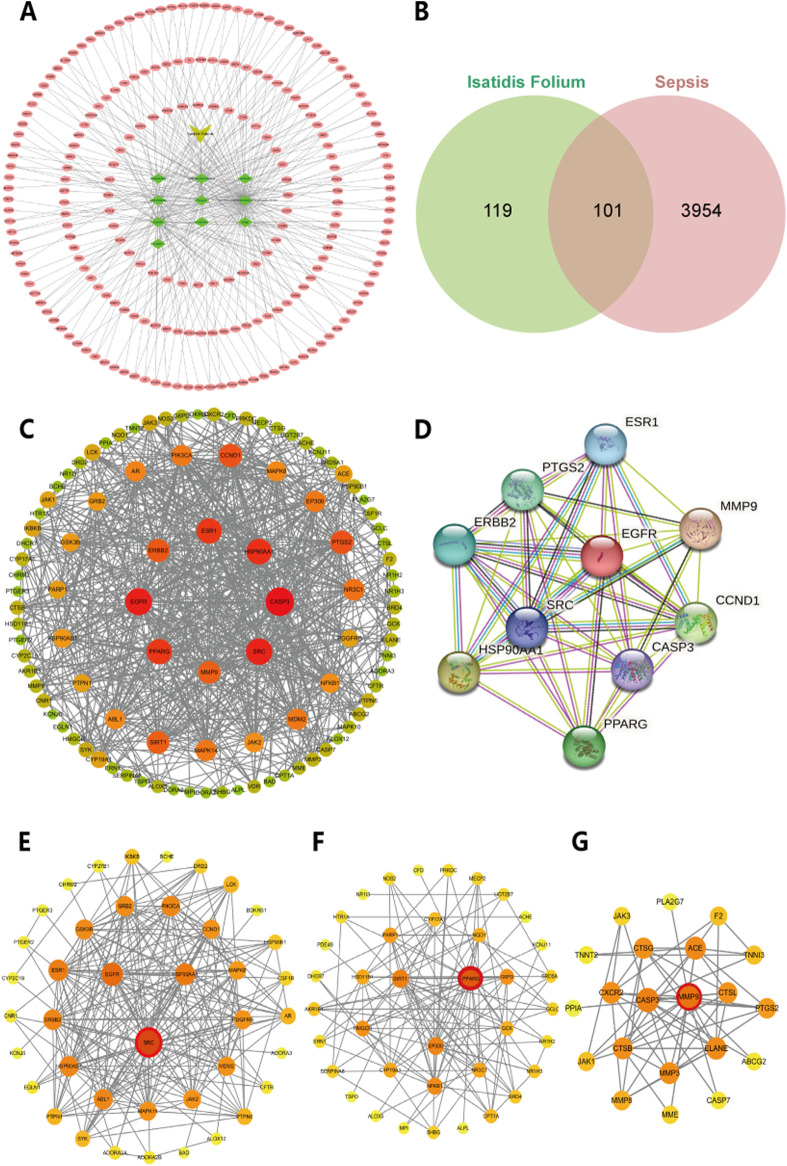
Network analysis of predicted targets for *Isatidis Folium* compounds and sepsis. **(A)** Potential targets for the active compounds in *Isatidis Folium*; **(B)** Venn diagram of active ingredients of *Isatidis Folium* and disease-related targets. **(C)** Protein-protein interaction (PPI) network diagram. The size of the node is proportional to the degree, and each edge represents the interaction between the compound molecule and the target. **(D)** The top 10 hub genes network. **(E–G)** Cluster analysis identified the top three core seed nodes involved in sepsis in the corresponding clusters. The seed nodes in each cluster were marked with red circles.

To identify the bioactive metabolites of *Isatidis Folium*, we conducted a virtual screening combining oral bioavailability (OB) and drug-likeness (DL) assessments using the TCMSP database. With screening threshold of OB ≥ 30% and DL ≥ 0.18, we identified 10 potential bioactive metabolites and 220 corresponding targets from the UniProt protein database (Supplementary Table S2). Using Cytoscape software, we constructed a systematic network of drug-metabolite-target interactions, analyzing the degree value of these metabolites to visually represent their associations with potential targets (Figure 1A). Based on degree values, the top five metabolites were identified as Indirubin (IDR), beta-sitosterol, poriferast-5-en-3beta-ol, 6-(3-oxoindolin-2-ylidene)indolo [2,1-b]quinazolin-12-one, and Glycyrol. Using the GeneCards database, we evaluated 4,055 potential targets associated with sepsis. The intersection between metabolite-related targets and disease-related targets was analyzed using Venny 2.1.0, revealing 101 shared targets, which are proposed to represent the potential therapeutic targets of *Isatidis Folium* for sepsis treatment (Figure 1B). These 101 targets were used to construct a protein-protein interaction (PPI) network, comprising 101 nodes and 713 edges, with an average node degree of 14.26 (Figure 1C). The top 10 therapeutic targets, ranked by weighted degree, were CASP3, EGFR, SRC, HSP90AA1, ESR1, PPARG, CCND1, PTGS2, ERBB2, SIRT1 (Figure 1D).

Cluster analysis using the MCODE plugin identified three distinct clusters, each represented by a seed node (Figures 1E–G; details in Table 2). The seed node of Cluster 1 is SRC (Figure 1E), a membrane-associated non-receptor tyrosine kinase known to mediate signaling pathways in LPS-stimulated macrophages (Ren et al., 2020). Inhibition of SRC activation can suppress downstream pathways, such as NF-κB and MAPK. The seed node of Cluster 2 is PPARG (Figure 1F), a peroxisome proliferator-activated receptor that suppresses the production of inflammatory factors, including IL-6, TNF-α, and IL-1β in monocytes, thereby modulating inflammation. The seed node of Cluster 3 is MMP9 (Figure 1G), a matrix metalloproteinase involved in inflammatory responses through extracellular matrix degradation and angiogenesis regulation. Inhibition of MMP9, either through gene knockout or pharmacological inhibitors, has been shown to attenuate inflammatory responses in sepsis and improve survival (Lorente et al., 2009; Hoffmann et al., 2009).

**TABLE 2:** Clusters of common targets.

Cluster	Number of edges	Number of nudes	Score
1	229	41	11.2
2	121	38	6.37
3	61	21	5.55

### 3.2 Target biological function analysis

**FIGURE 2:**
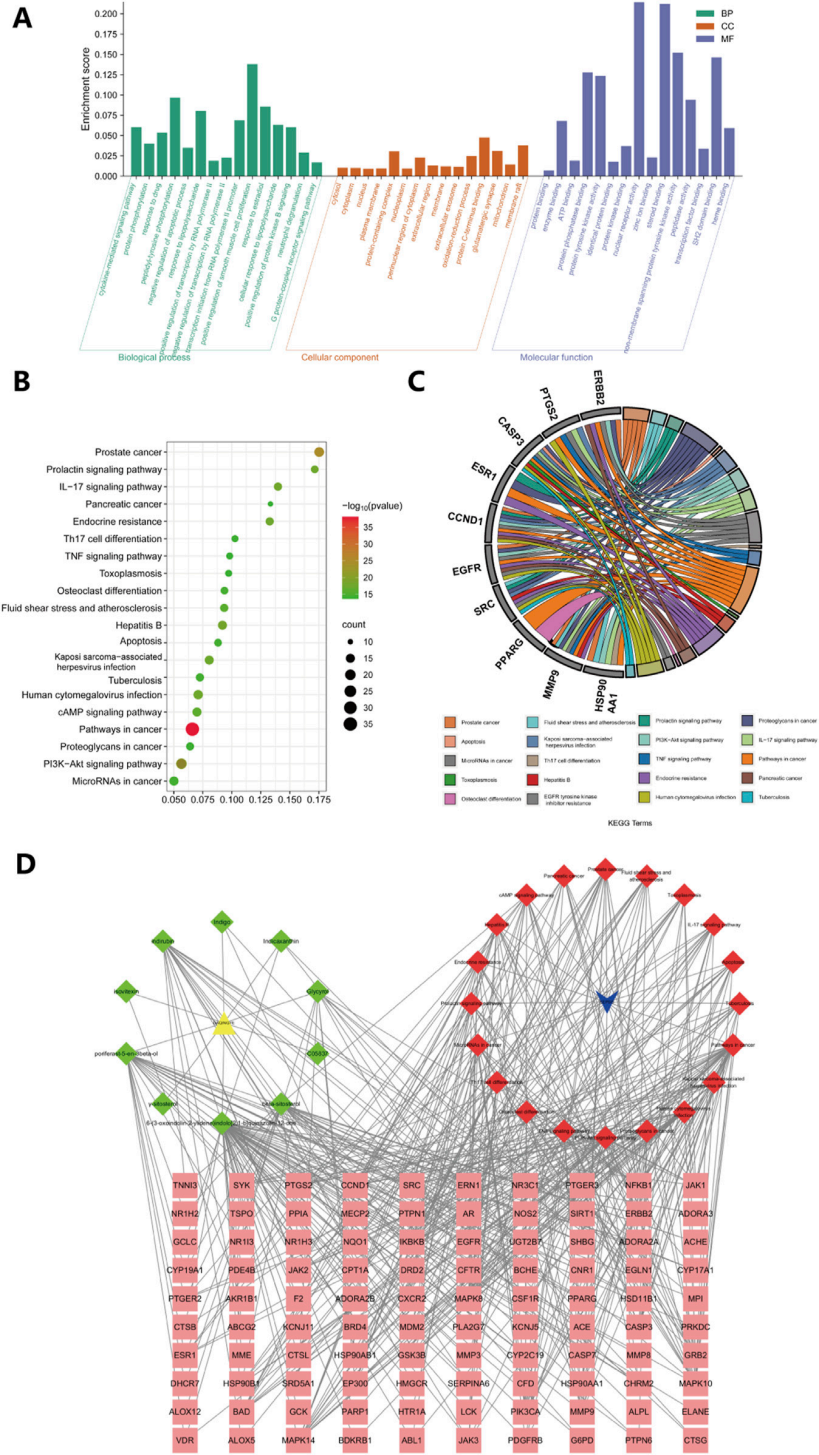
GO and KEGG enrichment analyses of 101 key targets. **(A)** Gene Ontology (GO) enrichment analysis of key targets in biological process (BP), cellular component (CC), and molecular function (MF). **(B)** Kyoto Encyclopedia of Genes and Genomes (KEGG) pathway enrichment analysis of key targets. **(C)** The top 20 pathways of hub genes. **(D)** Metabolite-disease-target-pathway network diagram for *Isatidis Folium*-mediated sepsis treatment. The pink squares in the diagram are the key targets of *Isatidis Folium* acting on the disease, the green quadrilaterals represent active compounds and the red quadrilaterals represents signaling pathways.

To gain a deeper understanding of the potential mechanisms by which *Isatidis Folium* influences sepsis, we conducted functional enrichment analyses of the 101 primary targets. Gene Ontology (GO) analysis revealed significantly enriched biological processes (BP), including response to lipopolysaccharide, cytokine-mediated signaling pathway, and protein phosphorylation; cellular components (CC), such as cytosol, cytoplasm, and nucleus; and molecular functions (MF), including protein binding, enzyme binding, and protein phosphatase binding (Figure 2A). Kyoto Encyclopedia of Genes and Genomes (KEGG) pathway analysis identified 230 significantly enriched pathways (p < 0.05), with the PI3K/Akt signaling pathway, IL-17 signaling pathway, and TNF signaling pathway being the most prominent (Figure 2B). Additionally, the hub genes most involved in these pathways were EGFR, SRC and CASP3 (Figure 2C). Network analysis of drug-target-pathway-disease interactions demonstrated that the therapeutic effects of *Isatidis Folium* on sepsis are mediated through multiple metabolites, targets, and pathways (Figure 2D).

Overall, these studies suggest that the bioactive metabolites of *Isatidis Folium* influence various biological processes, including cytokine production, protein phosphorylation, and response to lipopolysaccharide. Among these metabolites, degree value analysis identified Indirubin (IDR), beta-sitosterol, poriferast-5-en-3beta-ol, 6-(3-oxoindolin-2-ylidene)indolo [2,1-b]quinazolin-12-one, and Glycyrol as the five core metabolites, while CASP3, EGFR, SRC, HSP90AA1, and ESR1 were identified as the top five core targets. These targets were further analyzed through molecular docking.

### 3.3 Molecular docking verification

**FIGURE 3:**
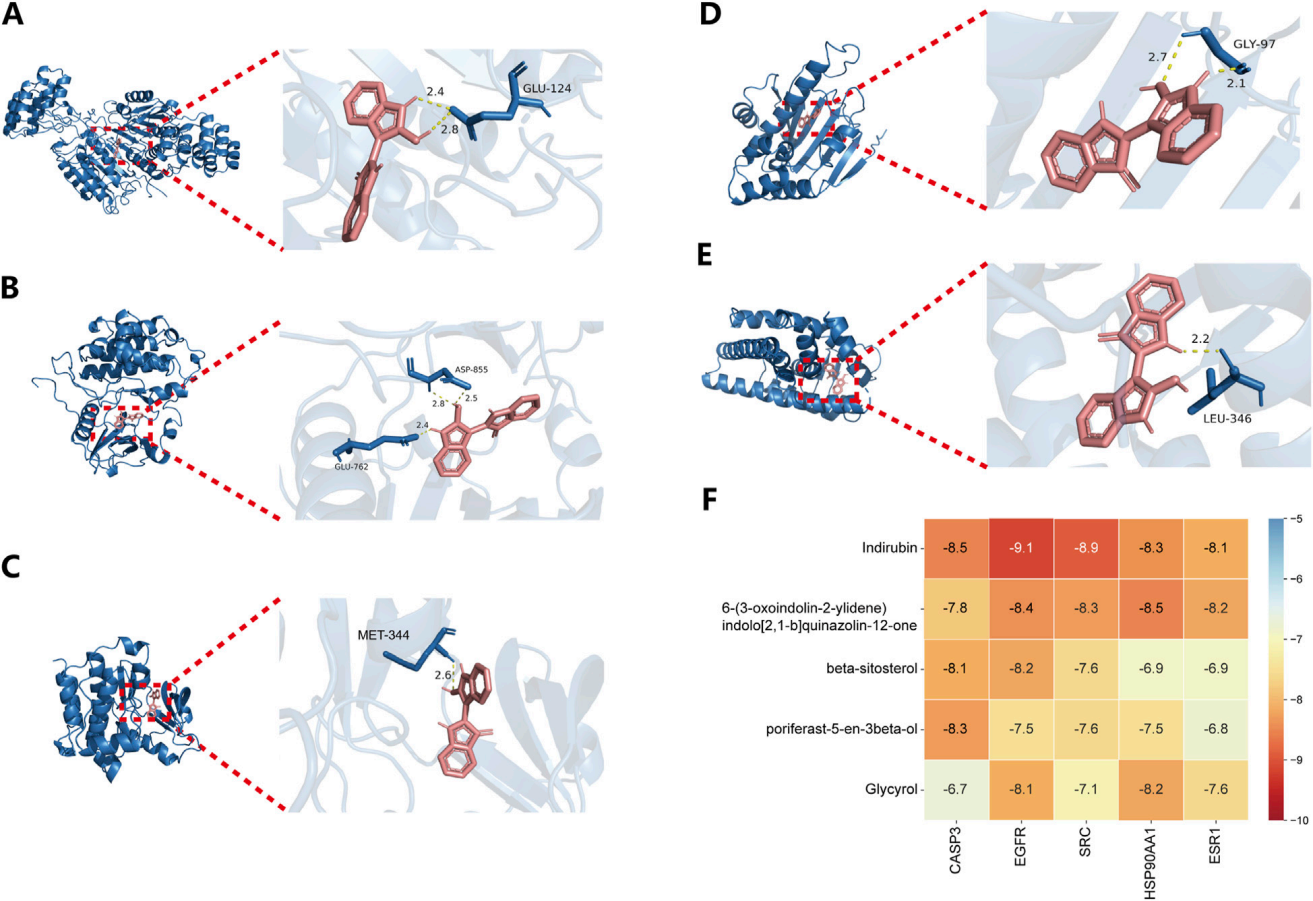
Results of key compound docking with key target molecules. Results of molecular docking of CASP3 **(A)**, EGFR **(B)**, SRC **(C)**, HSP90AA1 **(D)** and ESR1 **(E)** with Indirubin. **(F)** Heat maps of the docking binding energy of the top five compounds with the top five targets.

Based on the network analysis results, five metabolites and five targets were selected for molecular docking using AutoDockTools. Two-dimensional docking images were generated using Discovery Studio. All docking combinations exhibited binding energies below −4 kcal/mol, indicating strong binding affinity. A heatmap was used to visualize the binding energies of each configuration (Figure 3F). The results revealed that Indirubin exhibited higher binding affinity with potential targets compared to the other four metabolites, suggesting its role as a hub metabolite in *Isatidis Folium* for sepsis treatment. Notably, EGFR and SRC showed the highest affinity for Indirubin, with binding energies of −9.1 and −8.9 kcal/mol, respectively, suggesting their potential as critical therapeutic targets for sepsis. Three-dimensional docking images of Indirubin with the five targets are shown in Figures 3A–E, while additional docking results are provided in Supplementary Figure S1.

Based on the molecular docking results, we identified Indirubin as a high-ranking bioactive metabolite of *Isatidis Folium* with strong binding affinity to EGFR and SRC. Therefore, our subsequent experimental studies focused on these targets and Indirubin.

### 3.4 Impact of Indirubin on mice’s CLP-induced sepsis

**FIGURE 4:**
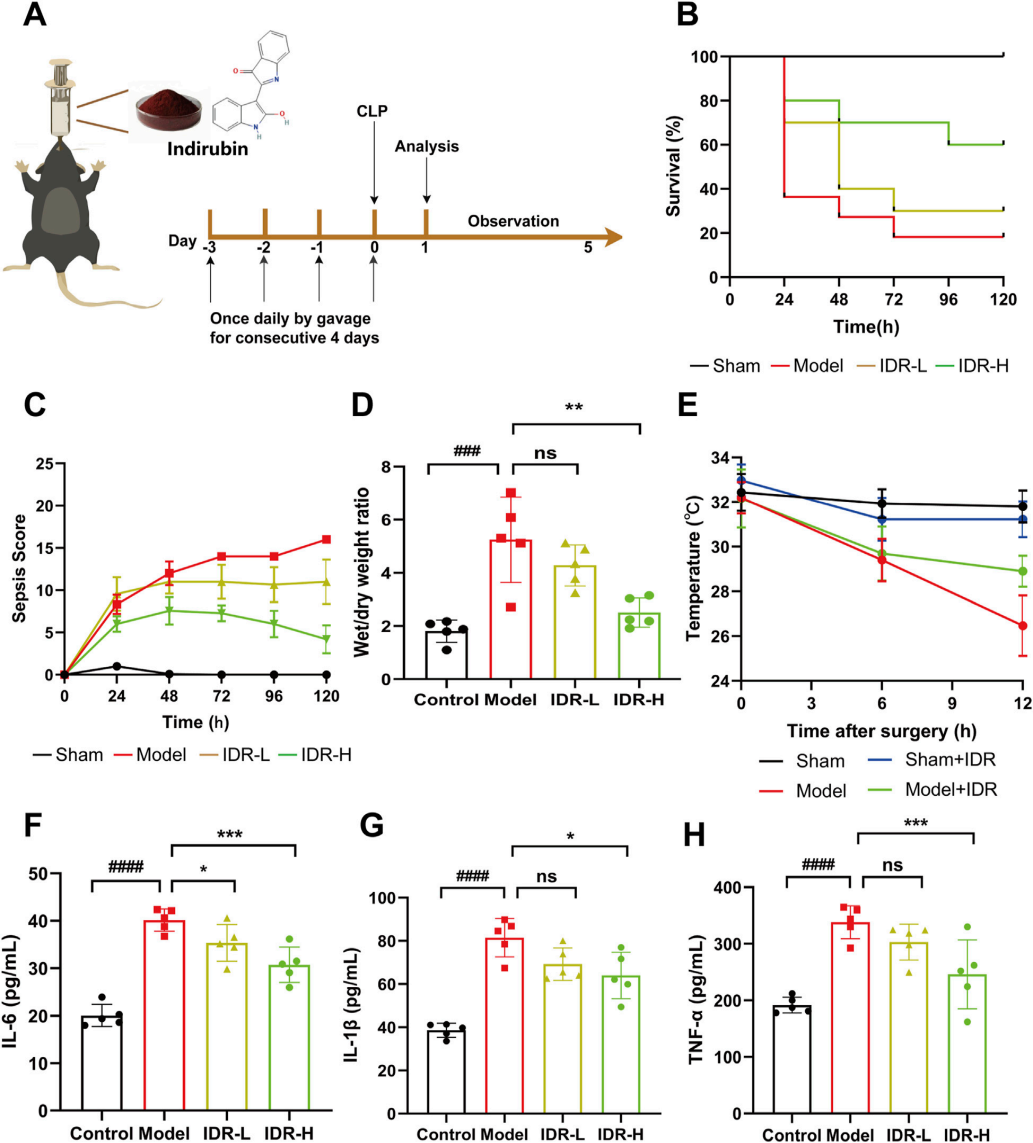
Indirubin attenuates inflammatory response in Cecal ligation and puncture (CLP)-Induced septic mice. **(A)** Schematic diagram of the pretreatment of Indirubin and establishment of animal mode *in vivo*. **(B)** The treatment effect of Indirubin on mortality with septic mice for 120 h (IDR-L: Indirubin low dose, 10 mg/kg. IDR-H: Indirubin high dose, 20 mg/kg). n = 10 per group. **(C)** The changes of murine sepsis score (MSS) in different groups within 120 h n = 10 per group. **(D)** The lung wet/dry ratio of mice was used to calculate the formation of pulmonary edema. n = 5 per group. **(E)** Body temperature change curves of mice in different treatment groups. n = 5 per group. ELISA detection of inflammatory cytokines, including IL-6 **(F)**, IL-1β **(G)**, and TNF-α **(H)** in the serum of different groups. n = 5 per group. The data are presented as mean ± SD. ^#^ *p* < 0.05, ^##^ *p* < 0.01, vs. Control group, *p < 0.05, **p < 0.01, ***p < 0.001 vs. Model group, ns = *p* > 0.05.

To evaluate the protective effects of Indirubin on cecal ligation and puncture (CLP)-induced sepsis in mice, we analyzed survival rates following administration of varying doses of Indirubin (Figure 4A). By day 5, all sham-operated mice survived, whereas only 20% of CLP-induced septic mice remained alive. Notably, pretreatment with Indirubin for 3 days significantly reduced mortality, with the high-dose group showing the most pronounced effect, confirming Indirubin’s protective role in acute septic shock (Figure 4B). These findings were corroborated by the murine sepsis score (MSS) (Figure 4C). Additionally, CLP-induced septic mice exhibited a higher lung wet/dry ratio compared to the sham group, indicating pulmonary edema. However, Indirubin treatment significantly reduced this ratio, demonstrating its ability to mitigate lung injury (Figure 4D). Body temperature, a critical indicator of sepsis severity and immune response, was also monitored (Chen et al., 2022b). The model group experienced a significant drop in body temperature (from 32°C to 26°C) within 12 h post-CLP surgery. In contrast, Indirubin treatment attenuated this decline (from 32°C to 30°C), highlighting its efficacy in improving hypothermia and stabilizing vital signs (Figure 4E).

Following the general observation, more comprehensive pathological investigations were later conducted. Sepsis is defined by the impairment of the immune system, which is closely linked to an overabundance of proinflammatory cytokines like TNF-α, IL-1β, and IL-6 (Angus and van der Poll, 2013). Inhibiting these cytokines has been shown to be an effective therapeutic strategy for sepsis (Willmann and Moita, 2024). As shown in Figures 4F–H, CLP surgery significantly increased serum levels of IL-1β, IL-6, and TNF-α, which were effectively reduced by Indirubin, confirming its broad anti-inflammatory properties.

**FIGURE 5:**
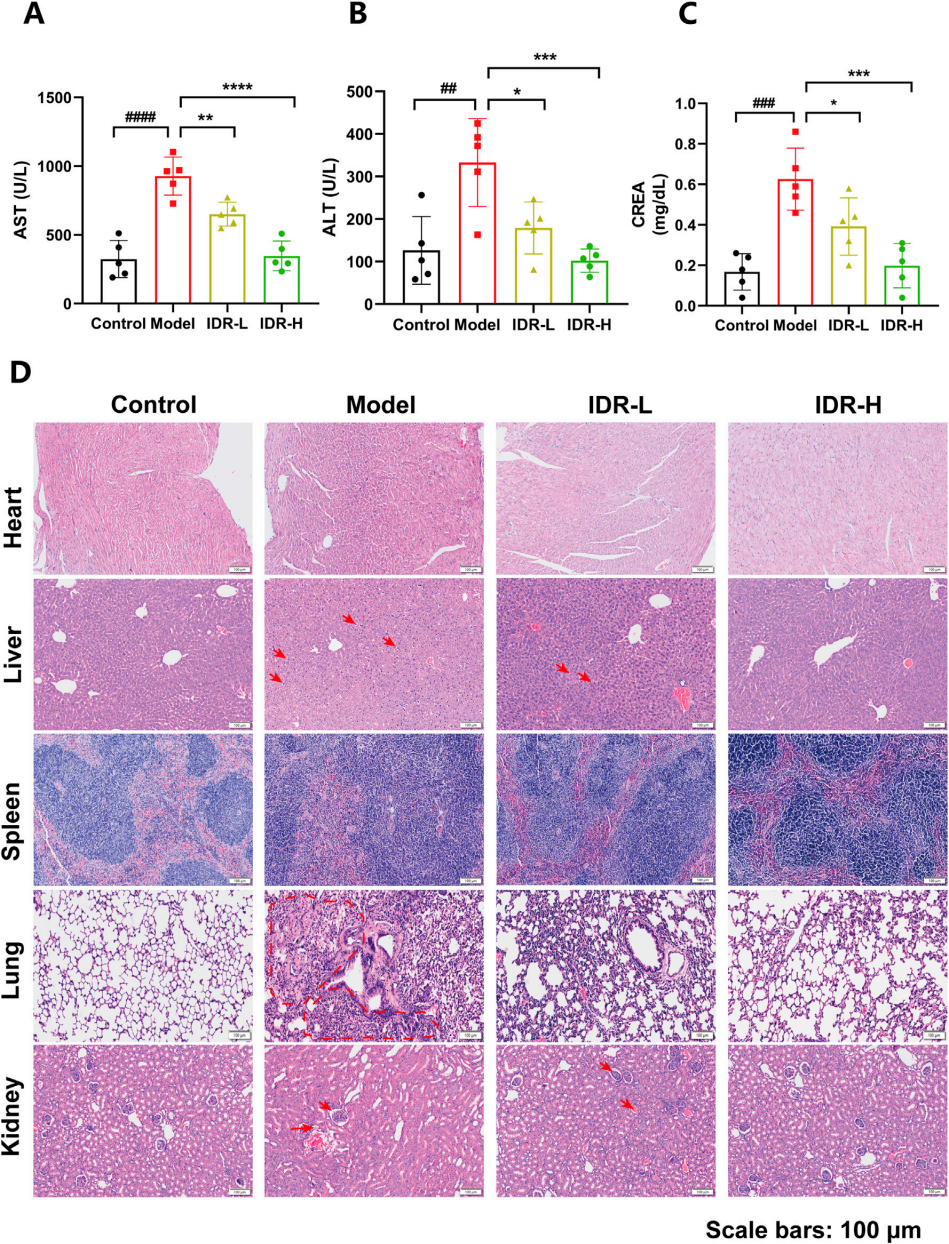
Protective effect of Indirubin treatment on multiple organs injury. Serum of AST **(A)**, ALT **(B)**, and CREA **(C)** in septic mice pretreated with different concentrations of Indirubin. n = 5 per group. **(D)** Hematoxylin and eosin (H&E) staining of heart, liver, spleen, lung, and kidney sections from different groups of mice (scale bar = 100 μM). The data are presented as mean ± SD. ^#^ *p* < 0.05, ^##^ *p* < 0.01 vs. Control group, *p < 0.05, **p < 0.01, ***p < 0.001 vs. Model group, ns = *p* > 0.05.

To assess organ damage, serum levels of alanine aminotransferase (ALT), aspartate aminotransferase (AST), and creatinine (CREA) were measured. Elevated levels of AST (Figure 5A), ALT (Figure 5B), and CREA (Figure 5C) in CLP-induced septic mice were significantly reduced by Indirubin, indicating improved liver and kidney function. Additionally, histopathological analysis via hematoxylin and eosin (H&E) staining further supported these findings. HE staining showed that mice treated with Indirubin had reduced the alveolar walls' thickening and inflammatory cells infiltration in the lungs. Additionally, there was a decline in the recruitment of inflammatory cells to blood vessels in the liver. The peripheral regions of the medulla surrounding the renal tissue in mice had tubular vacuoles, loss of brush border, and infiltration of inflammatory cells, together with vacuolation of histiocytes. These signs exhibited improvement in the groups treated with Indirubin, with the high-dose group demonstrating greater efficacy. This further reinforces the notion that Indirubin has protective effects on organ damage (Figure 5D).

In addition, Dexamethasone was used as a positive control group, and the expression of serum inflammatory factors was detected by ELISA, combined with biochemical analysis and histopathological analysis to observe the anti-inflammatory effect of Indirubin. The results showed that both Dexamethasone and Indirubin could significantly reduce the content of inflammatory factors in the serum of mice (Supplementary Figure S2), and there was no statistical difference between the two, which further confirmed the ideal effect of Indirubin in anti-inflammatory effect. In addition, all organs of mice treated with Dexamethasone and Indirubin basically returned to normal, and liver and kidney function indexes were also improved (Supplementary Figures S3, S4). These results further support the significant anti-inflammatory value and application potential of Indirubin.

In summary, Indirubin demonstrates extensive therapeutic benefits in the sepsis model by reducing pro-inflammatory cytokines, ameliorating hypothermia, and improving survival rates, further validating its therapeutic efficacy.

### 3.5 Indirubin suppresses the inflammatory response induced by LPS in RAW264.7

**FIGURE 6:**
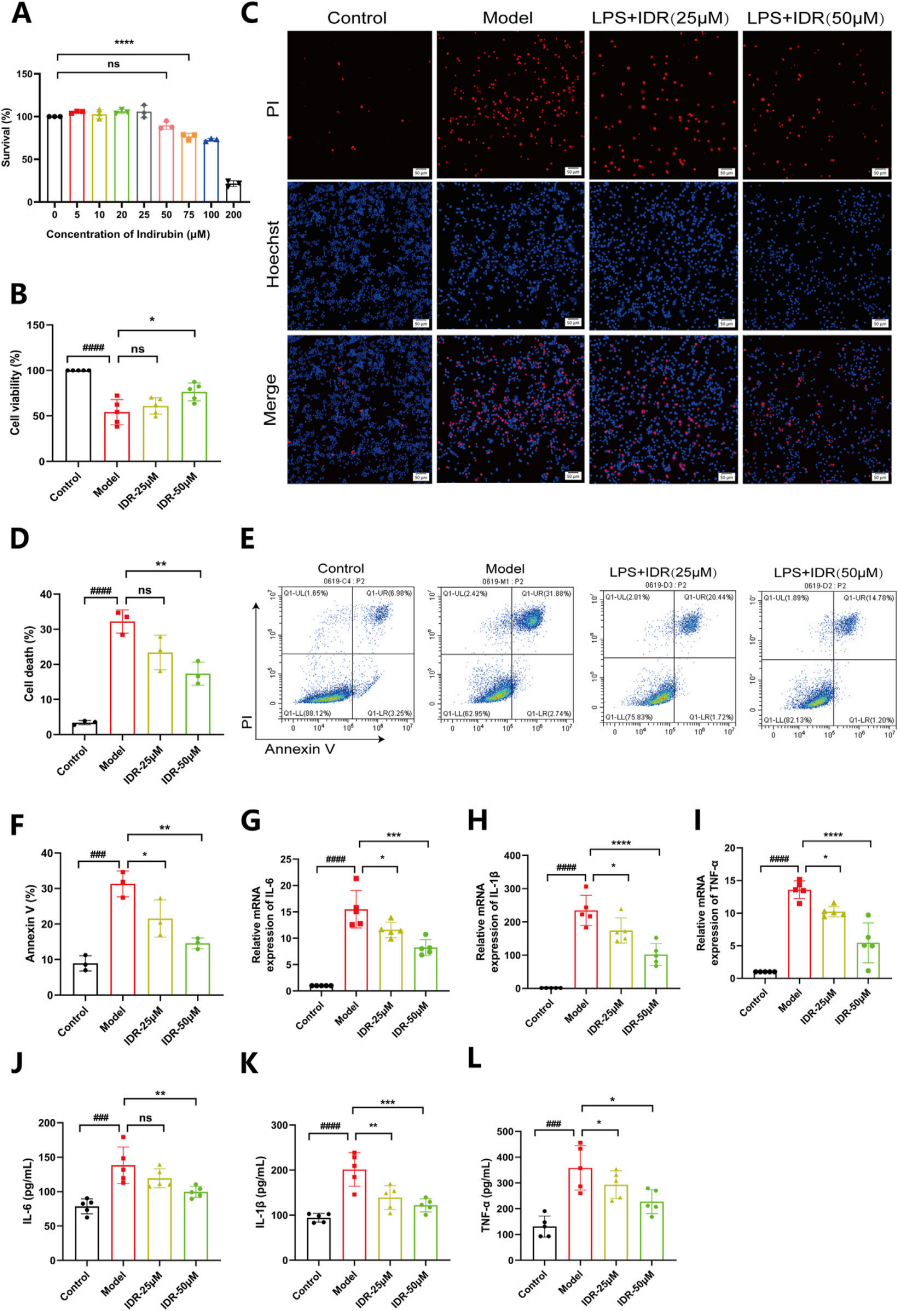
Indirubin suppresses macrophage death and inflammatory response induced by LPS. **(A)** Viability of RAW264.7 cells treated with different concentrations of Indirubin, respectively. n = 3 per group. **(B)** Viability of RAW264.7 cells treated with different concentrations of Indirubin followed by LPS (1 μg/mL) incubation, respectively. n = 3 per group. **(C, D)** Macrophage death was examined by PI/Hoechst staining and corresponding quantitative comparison of RAW264.7 cells exposed to different treatments, red fluorescence is for dead cells, the DAPI stained nucleus is blue, scale bar: 50 μm. **(E, F)** Flow cytometry analysis using Annexin V/PI staining and corresponding quantitative comparison of RAW264.7 cells treated with different concentrations of Indirubin. n = 3 per group. The mRNA expressions of IL-6 **(G)**, IL-1β **(H)** and TNF-α **(I)** in LPS-stimulated RAW264.7 cells upon Indirubin treatment were analyzed by qPCR. n = 5 per group. The secretion of inflammatory cytokines including IL-6 **(J)**, IL-1β **(K)** and TNF-α **(L)** was detected by ELISA. n = 5 per group. The data are presented as mean ± SD. ^#^ *p* < 0.05, ^##^ *p* < 0.01 vs. Control group, *p < 0.05, **p < 0.01, ***p < 0.001 vs. Model group, ns = *p* > 0.05.

Having validated the therapeutic effects of Indirubin *in vivo*, we next explored its ability to ameliorate inflammation *in vitro*. The MTT assay confirmed that Indirubin exhibited no cytotoxicity, even at high concentrations (50 μM), demonstrating excellent biocompatibility (Figure 6A). Taking advantage of its good biocompatibility, we further investigated the effect of Indirubin on LPS-induced cell death by using confocal laser scanning microscopy (CLSM). The findings, as shown in Figures 6C, D, demonstrated a notable rise in the quantity of dead cells (denoted by red signals) in the LPS-treated group (1 μg/mL). Conversely, after being treated with Indirubin, the number of dead cells was significantly reduced (as shown by red fluorescence). Furthermore, the results from CCK8 and flow cytometry also confirmed the protective capability of Indirubin in RAW264.7 cells (Figures 6B, E, F). According to Figure 6B, the cell damage induced by LPS was reduced greatly when RAW264.7 cells were pretreated with 50 μM Indirubin. The cell viability increased from 49.3% to 82.7%. Subsequently, using flow cytometry, we investigated the impact of Indirubin on LPS-induced cell death. As depicted in Figures 6E, F, the addition of Indirubin greatly reduced the percentage of dead cells caused by LPS treatment, demonstrating its cytoprotective capabilities.

We further evaluated Indirubin’s anti-inflammatory effects at the cellular level. RAW264.7 cells were stimulated with LPS (1 μg/mL) and treated with different concentrations of Indirubin. RT-PCR analysis revealed that LPS significantly upregulated IL-6, IL-1β, and TNF-α gene expression, which was effectively suppressed by Indirubin, with the high-dose group showing the strongest effect (Figures 6G–I). These results were further supported by ELISA, which demonstrated reduced levels of these cytokines in Indirubin-treated groups (Figures 6J–L). Similarly, we used Dexamethasone as a positive control and found that both Dexamethasone and Indirubin could significantly inhibit the expression of inflammatory factors in cells after LPS stimulation, with no statistical difference between them, further supporting the excellent anti-inflammatory potential of Indirubin (Supplementary Figure S5).

In conclusion, Indirubin protects RAW264.7 cells from LPS-induced cell death and reduces the expression of inflammatory factors.

### 3.6 Indirubin inhibited activation of the EGFR/SRC/PI3K and NF-κB/MAPK pathways

**FIGURE 7:**
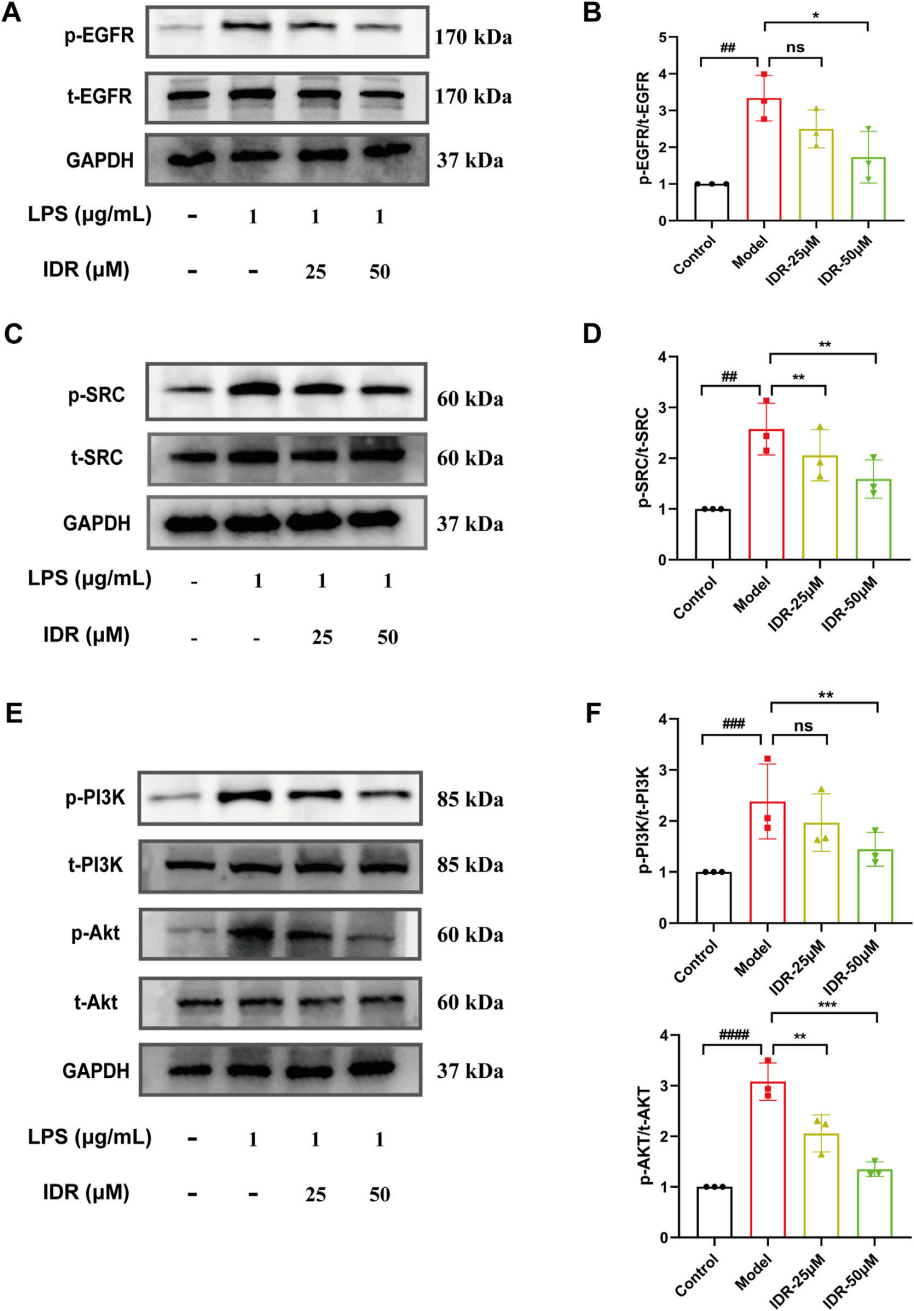
Indirubin inhibited activation of the EGFR/SRC/PI3K pathway. Western blot was used to detect the expression of p-EGFR/t-EGFR **(A, B)**, p-SRC/t-SRC **(C, D)**, p-AKT/t-AKT and p-PI3K/t-PI3K **(E, F)** in RAW264.7 cells. The data are presented as mean ± SD. ^#^ *p* < 0.05, ^##^ *p* < 0.01 vs. Control group, *p < 0.05, **p < 0.01, ***p < 0.001 vs. Model group, ns = *p* > 0.05.

Based on network pharmacology analysis of hub targets and pathway predictions, the anti-inflammatory effects of Indirubin, a bioactive metabolite of *Isatidis Folium*, appear to be mediated through the EGFR/SRC/PI3K signaling pathway. Molecular docking results indicated that SRC and EGFR are the most likely targets. Further GO and KEGG enrichment studies suggested that the PI3K/AKT signaling pathway plays a significant role in the protective effects of *Isatidis Folium* against sepsis. To validate these findings, we performed Western blot analysis to assess the impact of Indirubin on the expression of key proteins. The results showed a notable increase in the levels of phosphorylated SRC (p-SRC) and phosphorylated EGFR (p-EGFR) in LPS-activated RAW264.7 cells, while Indirubin treatment effectively reduced these levels (Figures 7A–D), consistent with network pharmacology predictions. These results highlight the critical role of the SRC and EGFR pathways in *Isatidis Folium* therapy for sepsis.

The PI3K-AKT pathway is traditionally known to regulate inflammatory responses (Vanhaesebroeck et al., 2012; Osaki et al., 2004). In this study, LPS stimulation markedly elevated the expression of phosphorylated PI3K (p-PI3K) and phosphorylated AKT (p-AKT) in RAW264.7 cells compared to controls. However, Indirubin intervention counteracted the activation, significantly reducing p-PI3K and p-AKT levels (Figures 7E, F), further supporting its anti-inflammatory role through the PI3K/AKT pathway. Together, these results suggest a potential role for EGFR/SRC/PI3K signaling pathway in attenuating the effects of Indirubin on sepsis.

**FIGURE 8:**
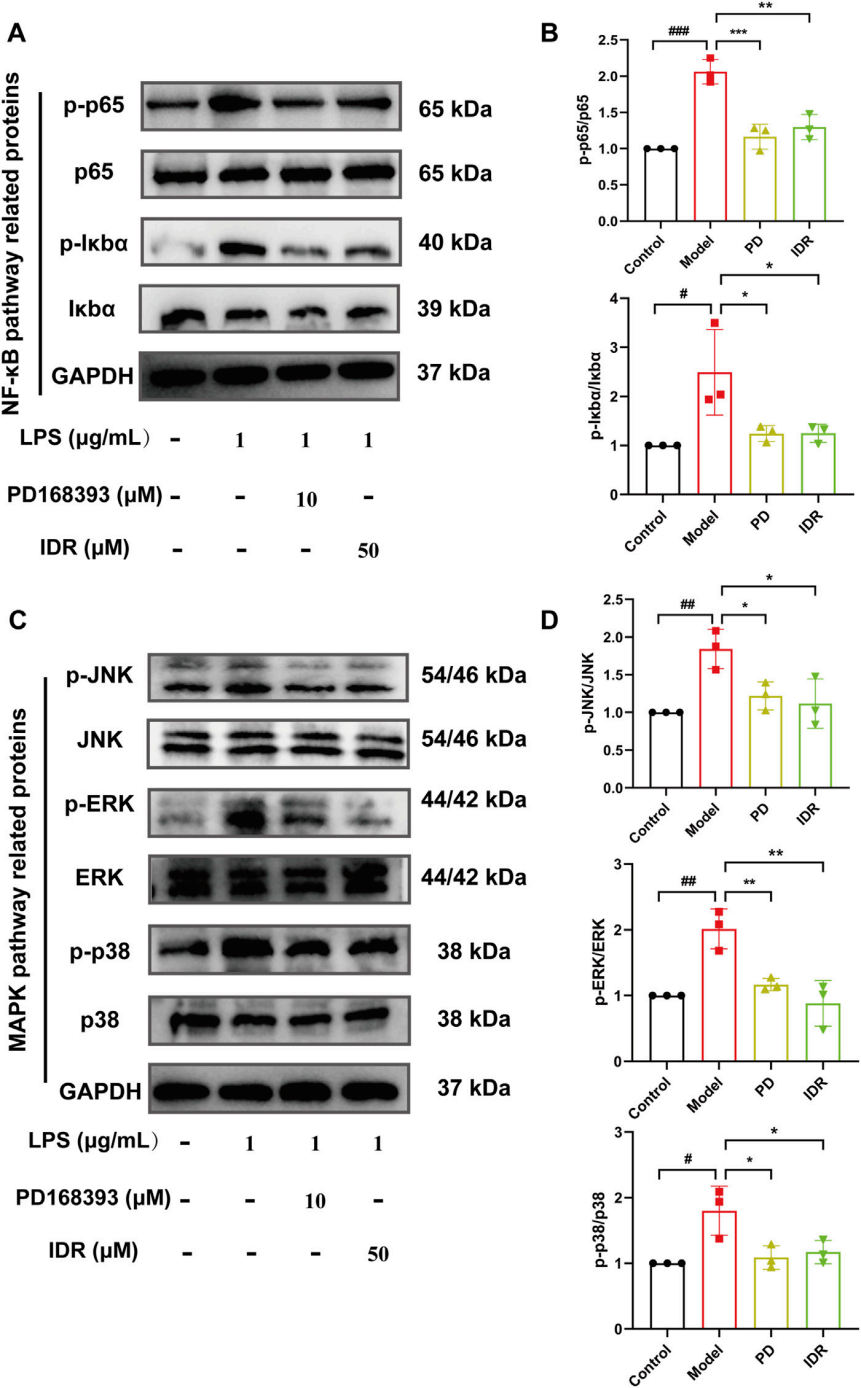
Indirubin inhibited activation of the NF-κB/MAPK pathway. Western blot was used to detect the expression of p-p65/p65, p-IκBα/IκBα **(A, B)**, p-JNK/JNK, p-ERK/ERK, and p-p38/p38 **(C, D)** in RAW264.7 cells. The data are presented as mean ± SD. ^#^p < 0.05, ^##^ *p* < 0.01, ^###^ *p* < 0.001 vs. Control group, *p < 0.05, **p < 0.01, ***p < 0.001 vs. Model group.

**FIGURE 9:**
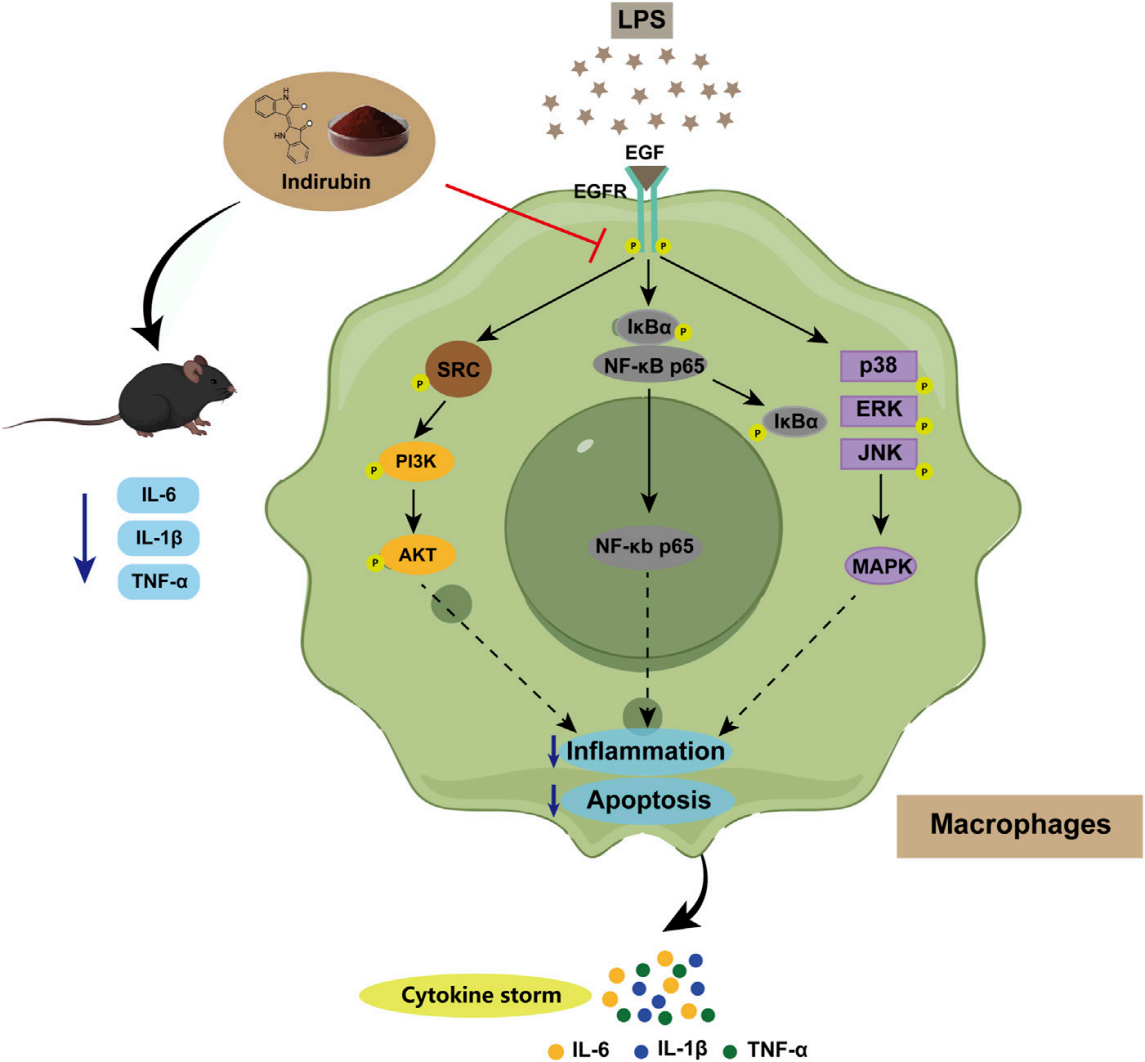
Schematic illustration of Indirubin alleviating sepsis by regulating the EGFR/SRC/PI3K and NF-κB/MAPK signaling pathways in macrophages.

Additionally, NF-κB and MAPK pathways are key mediators of LPS-induced inflammation. Previous studies, including those by Lai et al., have shown that Indirubin inhibits inflammation through NF-κB and MAPK signaling pathways (Yang et al., 2022; Liu et al., 2019; Lai et al., 2017b; Lai et al., 2017a; Wang et al., 2023). Furthermore, De et al. found that use of the EGFR inhibitor Erlotinib impaired the ability of Toll-like receptor 4 (TLR4) to activate NF-κB pathway in response to LPS, blocking the expression of LPS-induced inflammatory factors (De et al., 2015). zhang et al. have further shown that EGFR inhibition can attenuate inflammation mediated by the NF-κB/MAPK (Zhang et al., 2023). To validate this mechanism and confirm the central role of EGFR in this regulatory axis, we used the EGFR tyrosine kinase inhibitor PD168393 (10 μM) to block autophosphorylation at Tyr1068 (Zhang et al., 2023; Zhang et al., 2022; Li et al., 2014). Experimental data demonstrated that LPS stimulation significantly increased the phosphorylation of p65 and IκBα, activating the NF-κB pathway. Both Indirubin and PD168393 effectively inhibited p-p65 and p-IκBα expression, reduced p65 nuclear translocation, and suppressed NF-κB activation (Figures 8A, B). Similarly, Indirubin and PD168393 significantly inhibited the phosphorylation of ERK, JNK, and p38, key proteins in the MAPK pathway (Figures 8C, D). These results suggest that Indirubin can exert anti-inflammatory effects by inhibiting EGFR/SRC/PI3K and NF-κB/MAPK pathways (Figure 9).

### 3.7 Therapeutic effects of EGFR inhibitor on RAW264.7 cell and septic mice

To investigate whether Indirubin exerts its anti-inflammatory effects through EGFR, we used the EGFR tyrosine kinase inhibitor PD168393 at the cellular level. The results showed that the inflammatory response in the model group was significantly reduced after PD168393 treatment, meanwhile, we found that Indirubin could not further inhibit the inflammatory response of RAW264.7 cells in the presence of PD168393 (Supplementary Figure S6). This indicates that PD168393 abolishes the anti-inflammatory effects of Indirubin, suggesting that EGFR is a key mediator of Indirubin’s action. Furthermore, similar results were observed in animal models, where ELISA and histopathological analyses demonstrated that Indirubin’s anti-inflammatory effects on sepsis were attenuated by EGFR tyrosine kinase inhibitor Erlotinib (Supplementary Figures S4, S7). These findings collectively suggest that Indirubin likely exerts its anti-inflammatory effects on sepsis through the EGFR pathway.

### 3.8 Biocompatibility evaluations

Sepsis often triggers severe systemic inflammatory response syndrome (SIRS), which can lead to serious illness. If Indirubin shows toxicity *in vivo*, it may worsen the patient’s health condition. Therefore, we conducted a comprehensive biosafety evaluation of Indirubin through acute toxicity tests. We used different concentrations of Indirubin (10, 20, 40, 80 mg/kg) for three consecutive days. The body weight of the mice was followed for 7 days and the weight fluctuation pattern of the mice in the Indirubin-treated group was found to be consistent with that of the PBS control group (Supplementary Figure S8). In addition, the biosafety of Indirubin for major organs was evaluated by histological analysis. The results of H&E staining showed that the overall structure and integrity of the heart, liver, spleen, lung and kidney tissues of Indirubin-treated mice were not significantly different from those of normal mice (Supplementary Figure S9). Biochemical results further confirmed that even at the highest dose (80 mg/kg), Indirubin did not induce liver or kidney damage, as indicated by stable levels of liver and kidney function markers (Supplementary Figure S10). These findings suggest that Indirubin has an excellent biosafety profile and does not pose a risk of worsening health outcomes in animal models of sepsis.

## 4 Discussion

Sepsis is a major global health challenge characterized by high morbidity and mortality rates, often associated with multiple organ failure and dysregulated systemic inflammatory responses (Lelubre and Vincent, 2018). Currently, effective therapeutic options for sepsis remain limited. Traditional Chinese Medicine (TCM), with its extensive history and diverse herbal formulations, serves as a valuable resource for the development of novel therapeutic agents (Chao et al., 2017). Notably, *Isatidis Folium* has been reported to exhibit anti-endotoxic and antibacterial properties, highting its potential as a natural antibiotic (Deng et al., 2013). In this study, we identified Indirubin as the major bioactive metabolite of *Isatidis Folium* for sepsis treatment using network pharmacological analysis. Protein-protein interaction (PPI) network analysis revealed EGFR and SRC as the primary targets, suggesting their pivotal roles in sepsis therapy. Gene Ontology (GO) analysis indicated that *Isatidis Folium* primarily modulates biological process such as inflammatory response and apoptosis. Kyoto Encyclopedia of Genes and Genomes (KEGG) pathway analysis further suggested that *Isatidis Folium* targets the PI3K/AKT signaling pathway, which is critical for regulating inflammatory response.

Our experimental validation demonstrated that Indirubin effectively protected RAW264.7 cells from LPS-induced damage and significantly improved outcomes in a cecal ligation and puncture (CLP)-induced sepsis mouse model. To our knowledge, this is the first study to demonstrate the efficacy of Indirubin in CLP-induced sepsis. These findings suggest that Indirubin, the primary bioactive metabolite of *Isatidis Folium*, alleviates sepsis by mitigating inflammation through targeted modulation of key signaling pathways.

*Isatidis Folium* is a botanical drug with a long history of use in traditional Chinese medicine, spanning thousands of years. With advancements in modern pharmacology, an increasing number of pharmacological properties of *Isatidis Folium* have been identified (Muluye et al., 2014). For instance, Jiang et al. demonstrated that the n-butanol extract of *Isatidis Folium* exhibits potent anti-endotoxin activity both *in vitro* and *in vivo*. This extract significantly reduced inflammation and prevented infection by inhibiting the release of pro-inflammatory cytokines such as TNF-α and IL-6, thereby protecting mice from lung injuries associated with endotoxin-induced septic shock (Jiang et al., 2015). While previous studies have established the anti-inflammatory and therapeutic potential of *Isatidis Folium*, our study is the first to employ network pharmacology and molecular docking, combined with experimental validation, to identify specific bioactive metabolites and their potential targets for sepsis treatment. This approach provides a deeper understanding of the mechanisms underlying the therapeutic effects of *Isatidis Folium*.

Based on molecular docking results, we identified Indirubin, a bioactive metabolite of *Isatidis Folium*, as having strong binding affinity to SRC and EGFR, which guided our subsequent research focus. Xie et al. demonstrated that Indirubin reduces inflammatory responses by suppressing γδ T cells and inhibiting the Jak3/Stat3 pathway (Xie et al., 2018). Our experimental findings further support this, showing that Indirubin treatment significantly reduces inflammatory reactions in sepsis, aligning with the GO analysis results, which indicate that *Isatidis Folium* primarily targets inflammatory responses. In this study, EGFR and SRC were identified as key genes regulated by *Isatidis Folium*, with SRC also serving as a central hub in network cluster analysis. Both targets are well-documented for their critical roles in inflammatory response (Bian et al., 2024; El-Hashim et al., 2019). Furthermore, KEGG pathway analysis revealed that *Isatidis Folium* influences 225 pathways, particularly the PI3K-AKT pathway. EGFR, a multifunctional signaling molecule, regulates diverse cellular processes, including proliferation, migration, differentiation, inflammation, and stromal homeostasis. Upon ligand binding, EGFR activates multiple downstream signaling pathways, including the PI3K/AKT pathway (Wang et al., 2021). PI3K, a key component of the PI3K-AKT pathway, can be activated through various mechanisms to promote the release of pro-inflammatory cytokines and recruit inflammatory cells to tissues such as the lungs (Ding et al., 2020). Chen et al. reported that diregulin induces cartilage destruction in osteoarthritis by activating the EGFR/PI3K/AKT pathway (Chen et al., 2014), while AlaaeldinR et al. demonstrated that fatty acid glucoside inhibits inflammation by suppressing the EGFR/PI3K/AKT pathway (Alaaeldin et al., 2022). Additionally, SRC a downstream signaling molecule of EGFR, has been shown to play a pivotal role in macrophage-mediated inflammatory responses (Byeon et al., 2012). Based on these findings, we hypothesized that Indirubin, the primary bioactive metabolite of *Isatidis Folium,* exerts its anti-inflammatory effects in sepsis by modulating the EGFR/SRC/PI3K signaling pathway. Experimental results confirmed that Indirubin treatment significantly reduced the levels of PI3K and its downstream effector AKT in LPS-stimulated RAW264.7 cells. Furthermore, Indirubin decreased the phosphorylation of EGFR and SRC in LPS-activated RAW264.7 cells, suggesting that its anti-inflammatory effects in sepsis are mediated through the EGFR/SRC/PI3K pathway.

In addition, we observed that Indirubin exhibits significant inhibitory effects on the NF-κB/MAPK pathway (Lai et al., 2017b; Lai et al., 2017a). Concurrently, De et al. {De, 2015 #248} and Zhang et al. found that EGFR inhibition can attenuate inflammation mediated by the NF-κB/MAPK pathway (Zhang et al., 2023), prompting us to investigate whether Indirubin exerts its anti-inflammatory effects through EGFR inhibition. Here, we used the EGFR tyrosine kinase inhibitors PD168393 and Erlotinib for intervention. It was found that the levels of inflammatory factors in the model group were reduced after the use of inhibitors, while Indirubin could not further reduce the inflammatory response of RAW264.7 cells in the presence of inhibitors. This suggests that Indirubin likely mediates its anti-inflammatory effects through EGFR, further enriching our standing of its mechanism of action.

In this work, we demonstrated the potent anti-inflammatory effects of the primary bioactive metabolite of *Isatidis Folium* against LPS-induced inflammation *in vitro* and sepsis *in vivo*. By integrating network pharmacology with experimental validation, we elucidated the underlying mechanisms of action. Our findings provide robust evidence supporting the scientific rationale for the anti-inflammatory efficacy of *Isatidis Folium* and its bioactive metabolites. Sepecifically, we validated that Indirubin, the major bioactive metabolite of *Isatidis Folium,* exerts its therapeutic effects in sepsis by modulating the EGFR/SRC/PI3K and NF-κB/MAPK signaling pathways. These findings highlight Indirubin as a promising candidate for anti-inflammatory drug development. Moving forward, we plan to investigate other bioactive metabolites in *Isatidis Folium* to further elucidate their anti-inflammatory mechanisms.

Despite these findings, our study has several limitations. The mechanisms by which *Isatidis Folium* exerts its effects on sepsis are highly complex, and our study has only explored a subset of these pathways. For instance, additional signaling pathways that may be influenced by *Isatidis Folium* remain uninvestigated. Moreover, while we focused on specific bioactive metabolites of *Isatidis Folium*, it is possible that other undiscovered metabolites also contribute to its therapeutic efficacy. Additionally, while molecular docking provided valuable insights into the potential interactions between Indirubin and its targets (EGFR and SRC), this approach has inherent limitations which cannot definitively confirm direct binding interactions. To address this, future studies should employ experimental techniques such as surface plasmon resonance (SPR) or isothermal titration calorimetry (ITC) to validate the direct binding of Indirubin to EGFR and SRC. These experiments would provide more robust evidence to support our findings and further clarify the molecular mechanisms underlying Indirubin’s anti-inflammatory effects. Finally, although *Isatidis Folium* is generally well-tolerated, more comprehensive safety assessments are needed. Future studied should prioritize assessing the safety profile of this botanical drug in various patient populations, evaluating potential drug interactions, and identifying contraindications, particularly in the complex clinical context of sepsis.

## 5 Conclusion

This study employed network pharmacology combined with experimental validation to identify the bioactive metabolites and potential therapeutic targets of *Isatidis Folium* for sepsis treatment. We identified 10 primary bioactive metabolites and 220 potential targets associated with *Isatidis Folium* in sepsis. Molecular docking analysis further confirmed strong binding affinities between *Isatidis Folium* and hub targets. Both *in vitro* and *in vivo* experiments demonstrated that Indirubin, the major bioactive metabolite of *Isatidis Folium*, effectively mitigates sepsis-induced pathological damage and suppresses the production of pro-inflammatory molecules, like through inhibition of the EGFR/SRC/PI3K and NF-κB/MAPK signaling pathways. Our findings not only propose Indirubin as a promising therapeutic candidate for sepsis but also provide a comprehensive foundation for understanding the mechanisms underlying the therapeutic effects of *Isatidis Folium* in sepsis.

## Data Availability

The original contributions presented in the study are included in the article/Supplementary Material, further inquiries can be directed to the corresponding author.
