# Supplementary material for: Indirubin attenuates sepsis by targeting the EGFR/SRC/PI3K and NF-κB/MAPK signaling pathways in macrophages
Source: Front Pharmacol. 2025 Mar 12;16:1542061. doi: 10.3389/fphar.2025.1542061 (PMC11938131; doi:10.3389/fphar.2025.1542061)
Supplement: Supplementary file 1 [file DataSheet1.pdf]

Indirubin Attenuates Sepsis by Targeting the EGFR/SRC/PI3K and NF-κB/MAPK Signaling pathways in Macrophages

Table S1. List of antibodies used for WB.

Antibodies	Suppliers (catalog number)	Antibodies	Suppliers (catalog number)
EGFR	Cell Signaling Technology (2232)	p-EGFR	Cell Signaling Technology (2234)
Src	Cell Signaling Technology (2108)	p-Src	Cell Signaling Technology (2101)
PI3K	Cell Signaling Technology (4292)	p-PI3K	Cell Signaling Technology (4228)
Akt	Cell Signaling Technology (9272)	p-Akt	Cell Signaling Technology (9271)
IκBα	Cell Signaling Technology (4812)	p-IκBα	Cell Signaling Technology (2859)
p65	Cell Signaling Technology (8242)	p-p65	Cell Signaling Technology (3033)
ERK	Cell Signaling Technology (4695)	p-ERK	Cell Signaling Technology (4370)
JNK	Wanlei Biotechnology (WL01295)	p-JNK	Wanlei Biotechnology (WL01813)
p38	Wanlei Biotechnology (WL00764)	p-p38	Wanlei Biotechnology (WL03428)
GAPDH	Cell Signaling Technology (2118)	β -actin	Cell Signaling Technology (4970)
Goat anti-rabbit IgG-HRP	Proteintech (SA00001-2)	Goat anti-mouse IgG-HRP	Proteintech (SA00001-1)

Table S2. Information table of active ingredients of *Isatidis Folium*.

**ID**	**Compound**	**2D Structure**	**Degree**	**Formula**	**MW（g/mol）**
1	Indirubin	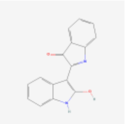	71	$C_{16}H_{10}N_2O_2$	262.28
2	6-(3-oxoindolin-2-ylidene) indolo [2,1-b]quinazolin-12-one	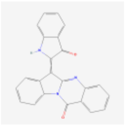	65	$C_{23}H_{13}N_3O_2$	363.39
3	beta-sitosterol	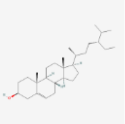	44	$C_{29}H_{50}O$	414.79
4	poriferast-5-en-3beta-ol	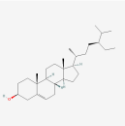	41	$C_{29}H_{50}O$	414.79

5	Glycyrol	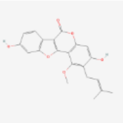	21	$C_{21}H_{18}O_6$	366.39
6	Indigo	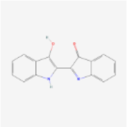	17	$C_{16}H_{10}N_2O_2$	262.28
7	Indicaxanthin	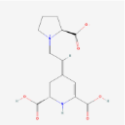	13	$C_{14}H_{16}N_2O_6$	308.32
8	isovitexin	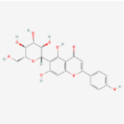	4	$C_{21}H_{20}O_{10}$	432.41
9	γ-sitosterol	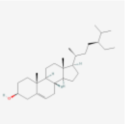	3	$C_{29}H_{50}O$	414.79
10	C05827	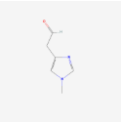	2	$C_{16}H_{20}N_2O_9S_2$	448.52

Table S3. Murine sepsis scoring criteria.

**Variable**	**Score and description**
Appearance	0—Coat is smooth<br>1—Patches of hair piloerected<br>2—Majority of back is piloerected<br>3—Piloerection may or may not be present, mouse appears "puffy"<br>4—Piloerection may or may not be present, mouse appears emaciated
Level of consciousness	0—Mouse is active<br>1—Mouse is active but avoids standing upright<br>2—Mouse activity is noticeably slowed. The mouse is still ambulant<br>3—Activity is impaired. Mouse only moves when provoked, movements have a tremor<br>4—Activity severely impaired. Mouse remains stationary when provoked, with possible tremor
Activity	0—Normal amount of activity. Mouse is any of: eating, drinking, climbing, running, fighting<br>1—Slightly suppressed activity. Mouse is moving around bottom of cage<br>2—Suppressed activity. Mouse is stationary with occasional investigative movements<br>3—No activity. Mouse is stationary<br>4—No activity. Mouse experiencing tremors, particularly in the hind legs

Response to stimulus	0—Mouse responds immediately to auditory stimulus or touch<br>1—Slow or no response to auditory stimulus; strong response to touch (moves to escape)<br>2—No response to auditory stimulus; moderate response to touch (moves a few steps)<br>3—No response to auditory stimulus; mild response to touch (no locomotion)<br>4—No response to auditory stimulus. Little or no response to touch. Cannot right itself if pushed over
Eyes	0—Open<br>1—Eyes not fully open, possibly with secretions<br>2—Eyes at least half closed, possibly with secretions<br>3—Eyes half closed or more, possibly with secretions<br>4—Eyes closed or milky
Respiration rate	0—Normal, rapid mouse respiration<br>1—Slightly decreased respiration (rate not quantifiable by eye)<br>2—Moderately reduced respiration (rate at the upper range of quantifying by eye)<br>3—Severely reduced respiration (rate easily countable by eye, 0.5 s between breaths)<br>4—Extremely reduced respiration (> 1 s between breaths)
Respiration quality	0—Normal<br>1—Brief periods of laboured breathing<br>2—Laboured, no gasping<br>3—Laboured with intermittent gasps<br>4—Gasping

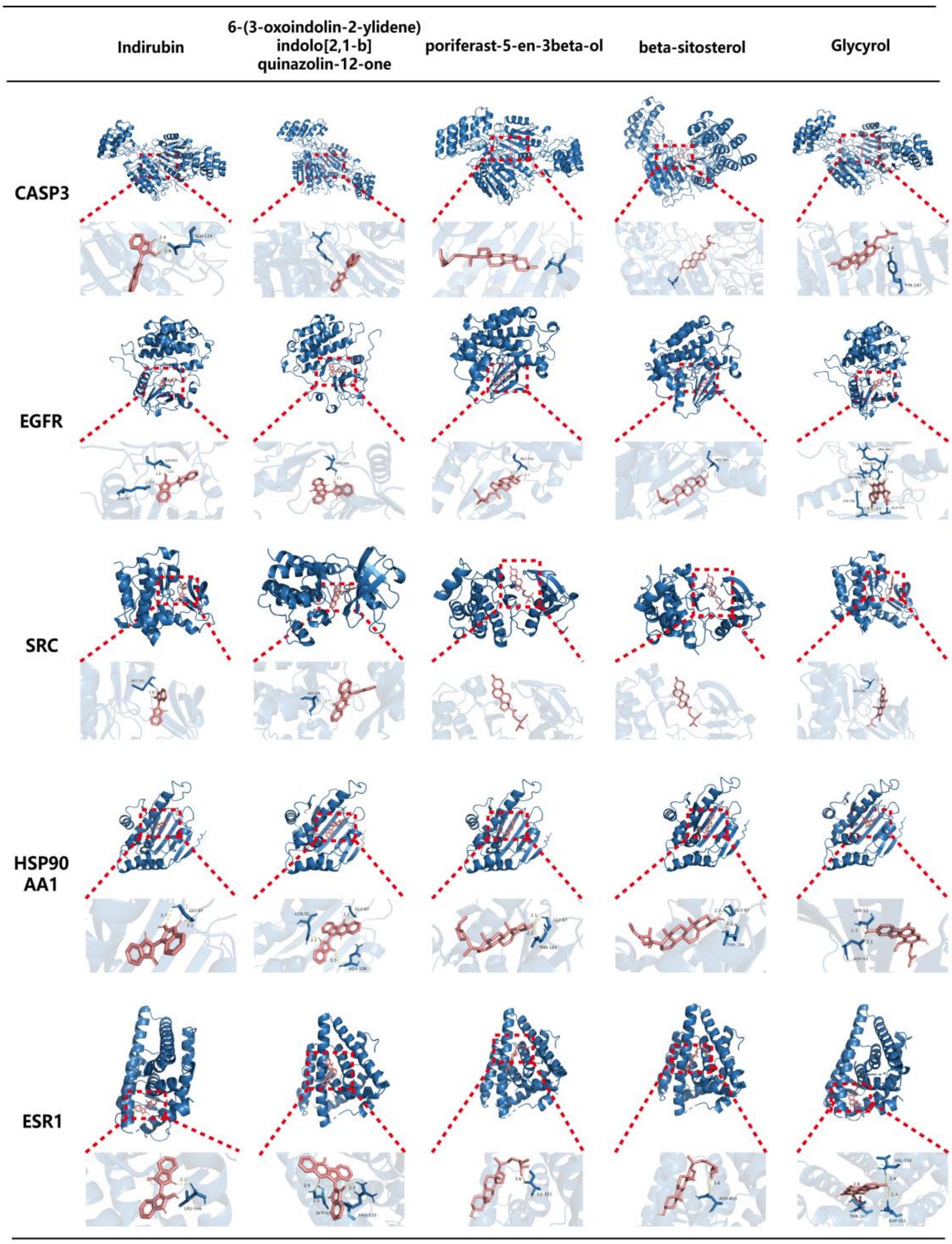

Figure S1. Results of molecular docking of the top five compounds with the top five targets.

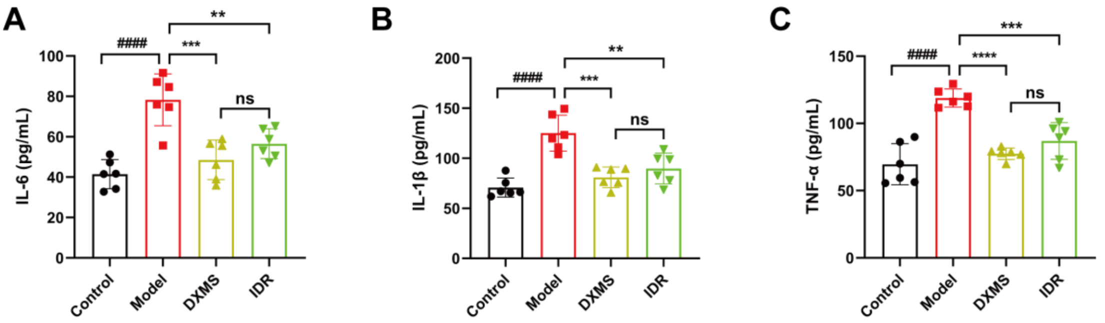

Figure S2. ELISA detection of inflammatory cytokines, including IL-6, IL-1β, and TNF-α, in the serum of different groups (DXMS: Dexamethasone, 10 mg/kg; IDR: Indirubin, 20 mg/kg). n=6 per group. The data are presented as mean ± SD. ####$p < 0.0001$, vs. Control group, **$p < 0.01$, ***$p < 0.001$, ****$p < 0.0001$ vs. Model group, ns = $p > 0.05$.

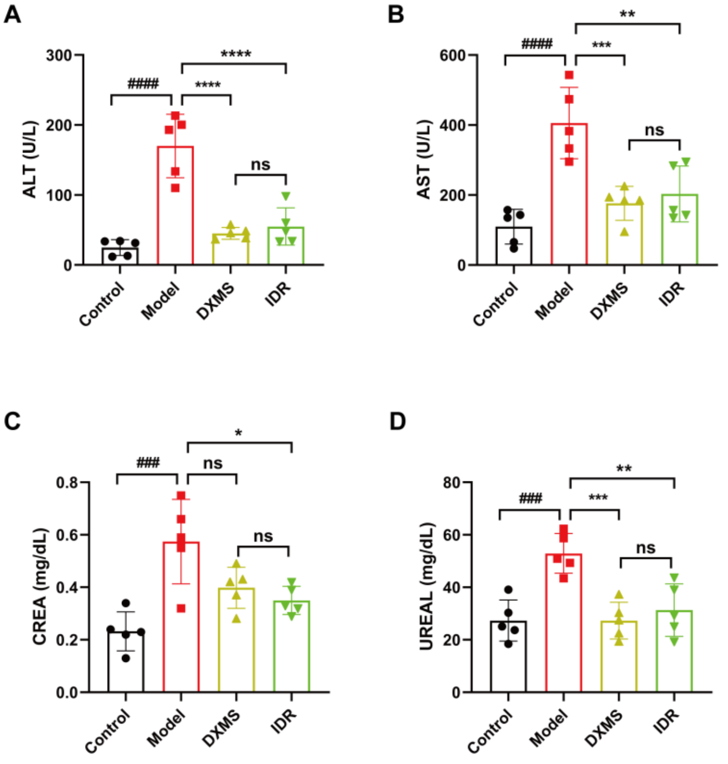

Figure S3. Serum of ALT (A), AST (B), CREA (C), and UREAL (D) in septic mice pretreated with Dexamethasone (DXMS, Dexamethasone, 10 mg/kg) and Indirubin (IDR, 20 mg/kg). n=5 per group. The data are presented as mean ± SD. ###$p < 0.001$, ####$p < 0.0001$, vs. Control group, *$p < 0.05$, **$p < 0.01$, ***$p < 0.001$, ****$p < 0.0001$ vs. Model group, ns = $p > 0.05$.

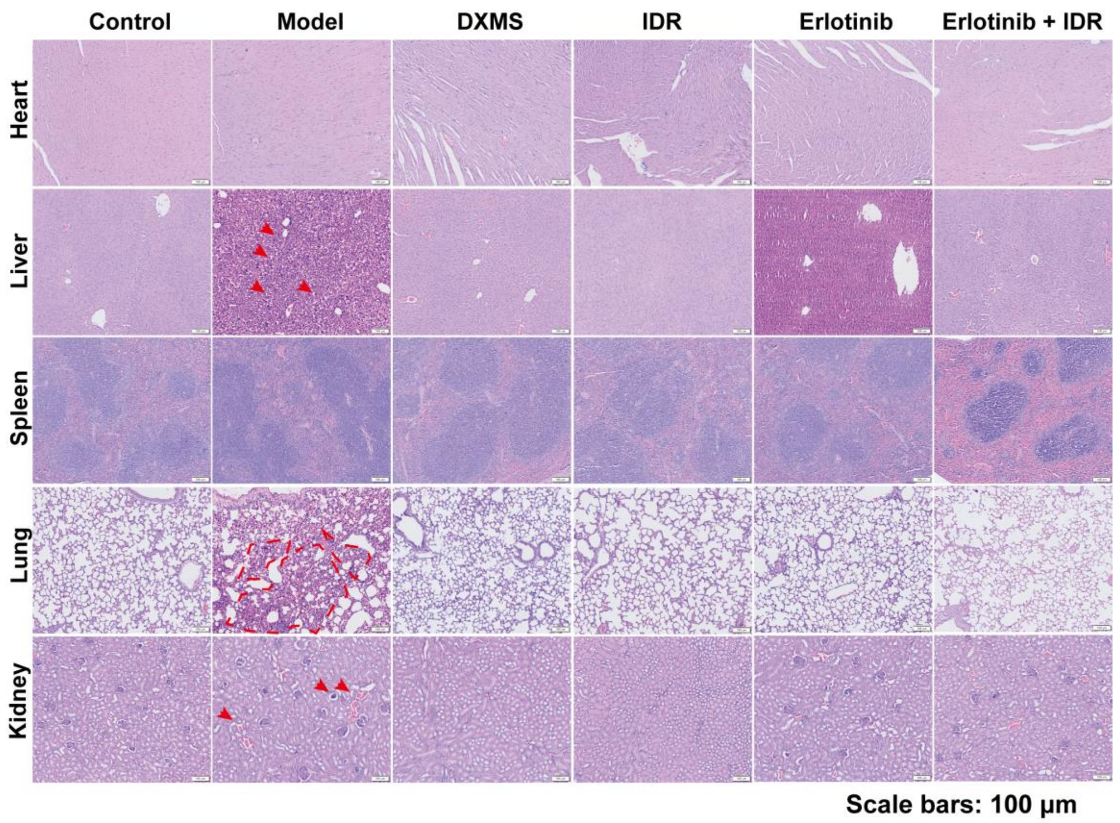

Figure S4. Hematoxylin and eosin (H&E) staining of heart, liver, spleen, lung, and kidney sections from different groups of mice (DXMS, Dexamethasone, 10 mg/kg; IDR: Indirubin, 20 mg/kg; Erlotinib: 45 mg/kg; Erlotinib + IDR, Erlotinib 45 mg/kg, IDR, 20 mg/kg), (scale bar = 100 μm).

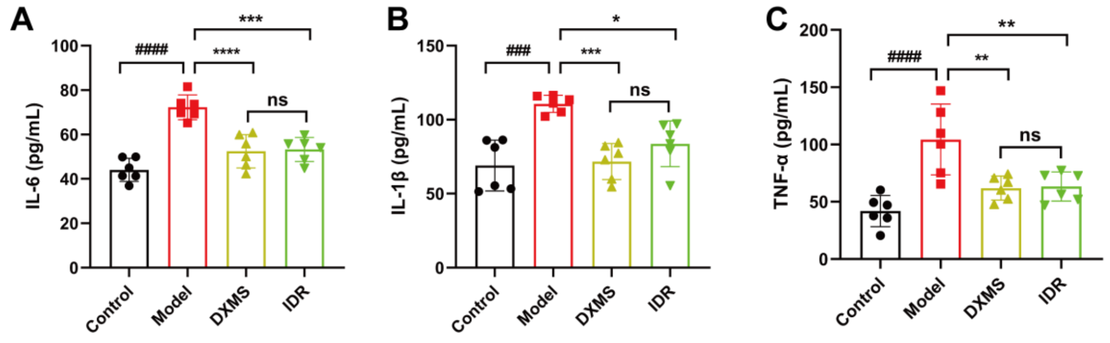

Figure S5. The secretion of inflammatory cytokines including IL-6 (A), IL-1β (B) and TNF-α (C) was detected by ELISA, (DXMS: Dexamethasone, 10 μM; IDR: Indirubin, 50 μM). n=5 per group. The data are presented as mean ± SD. ###$p < 0.05$, ####$p < 0.01$ vs. Control group, *$p < 0.05$, **$p < 0.01$, ***$p < 0.001$, ****$p < 0.0001$ vs. Model group, ns = $p > 0.05$.

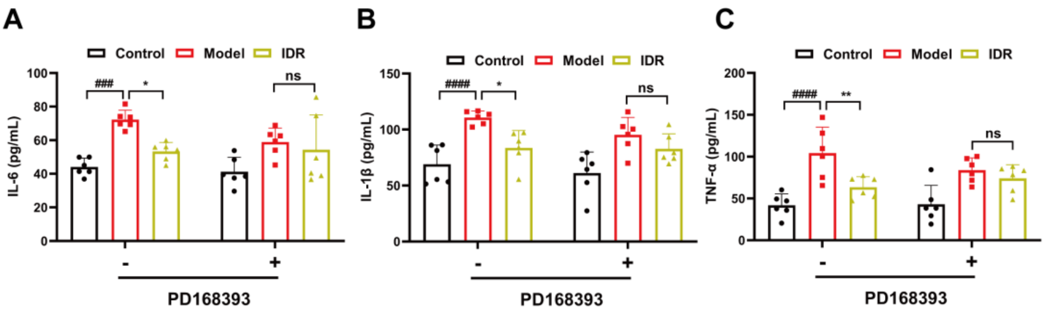

Figure S6. The secretion of inflammatory cytokines including IL-6 (A), IL-1β (B) and TNF-α (C) was detected by ELISA (PD168393: 10 μM; IDR: Indirubin, 50 μM). n=5 per group. The data are presented as mean ± SD. $^{\#\#\#}p < 0.05$, $^{\#\#\#\#}p < 0.01$ vs. Control group, $^{*}p < 0.05$, $^{**}p < 0.01$ vs. Model group, ns = $p > 0.05$.

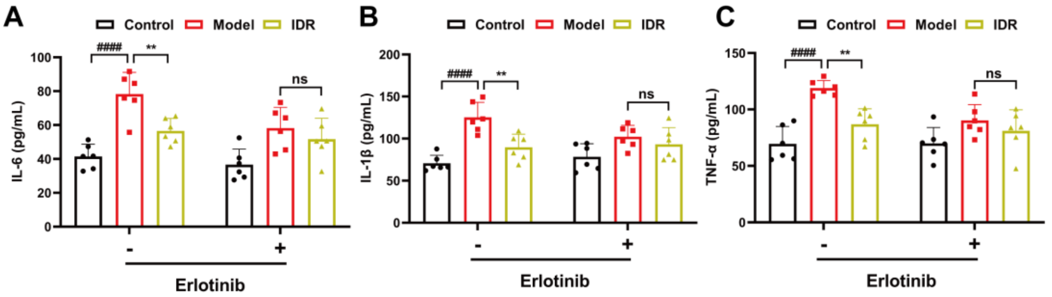

Figure S7. ELISA detection of inflammatory cytokines, including IL-6 (A), IL-1β (B), and TNF-α (C), in the serum of different groups (Erlotinib: 45 mg/kg; IDR: Indirubin, 20 mg/kg). n=6 per group. The data are presented as mean ± SD. $^{\#\#\#\#}p < 0.0001$, vs. Control group, $^{**}p < 0.01$, $^{***}p < 0.001$, $^{****}p < 0.0001$ vs. Model group, ns = $p > 0.05$.

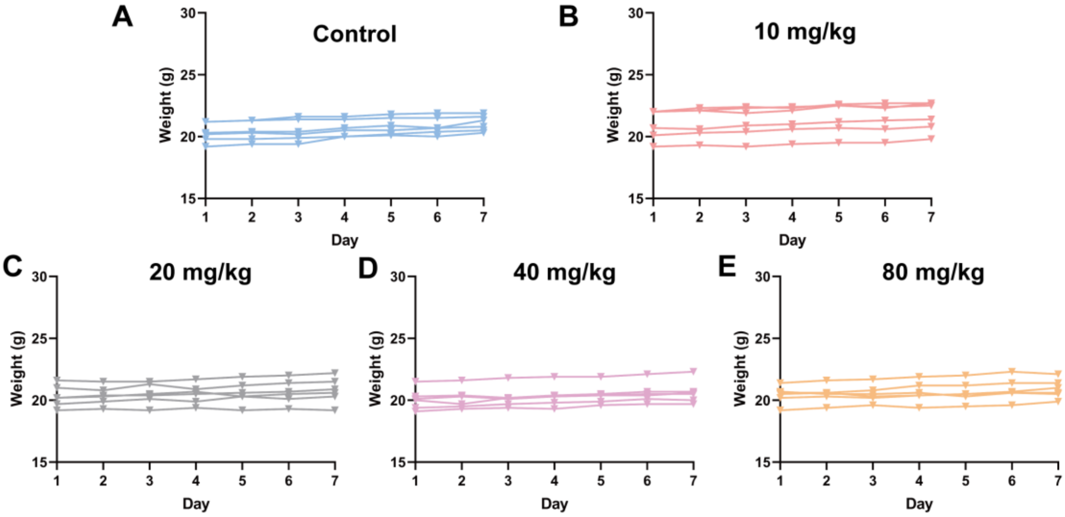

Figure S8. Body weight of mice in different treatment groups recorded for 7 days, respectively.

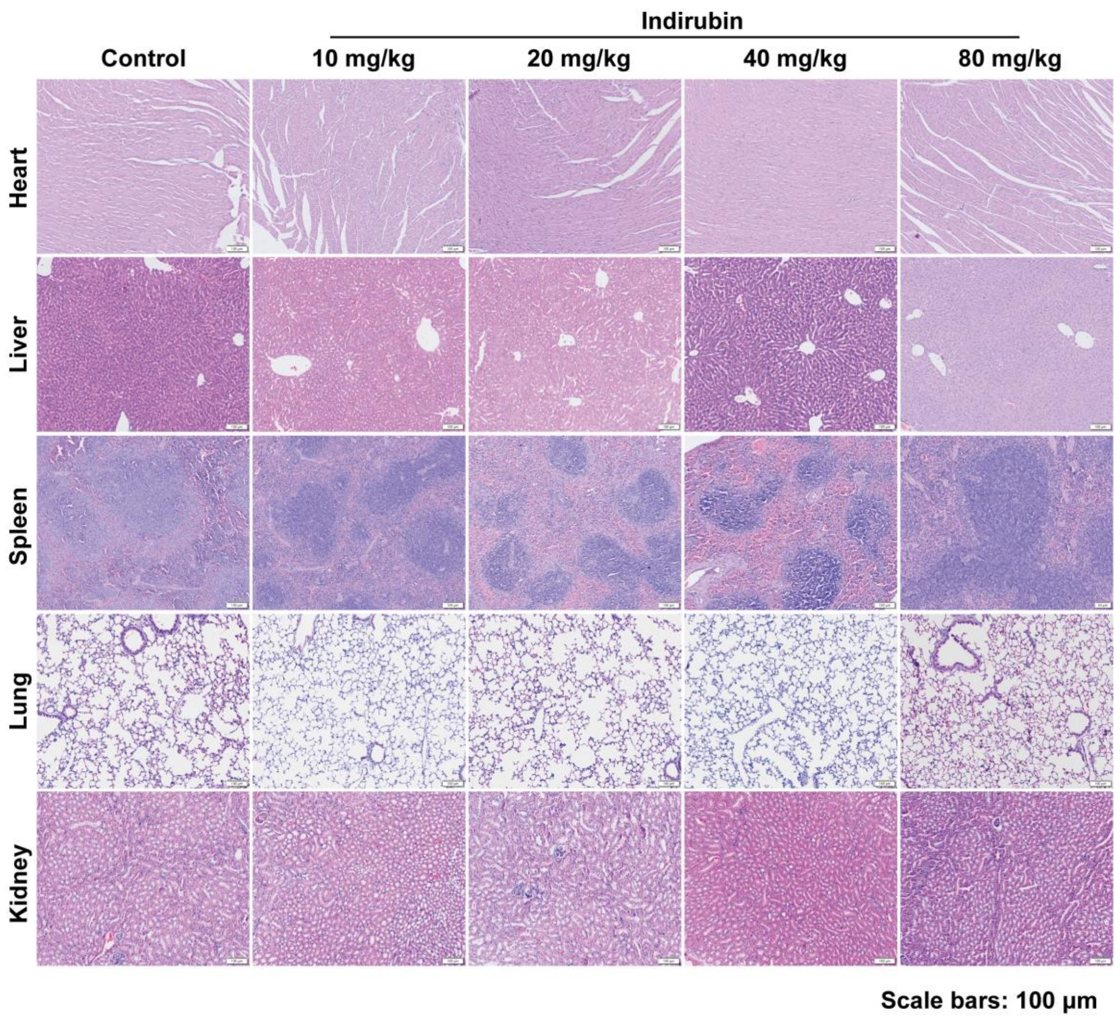

Figure S9. Hematoxylin and eosin (H&E) staining of heart, liver, spleen, lung, and kidney sections from different groups of mice (scale bar = 100 μm).

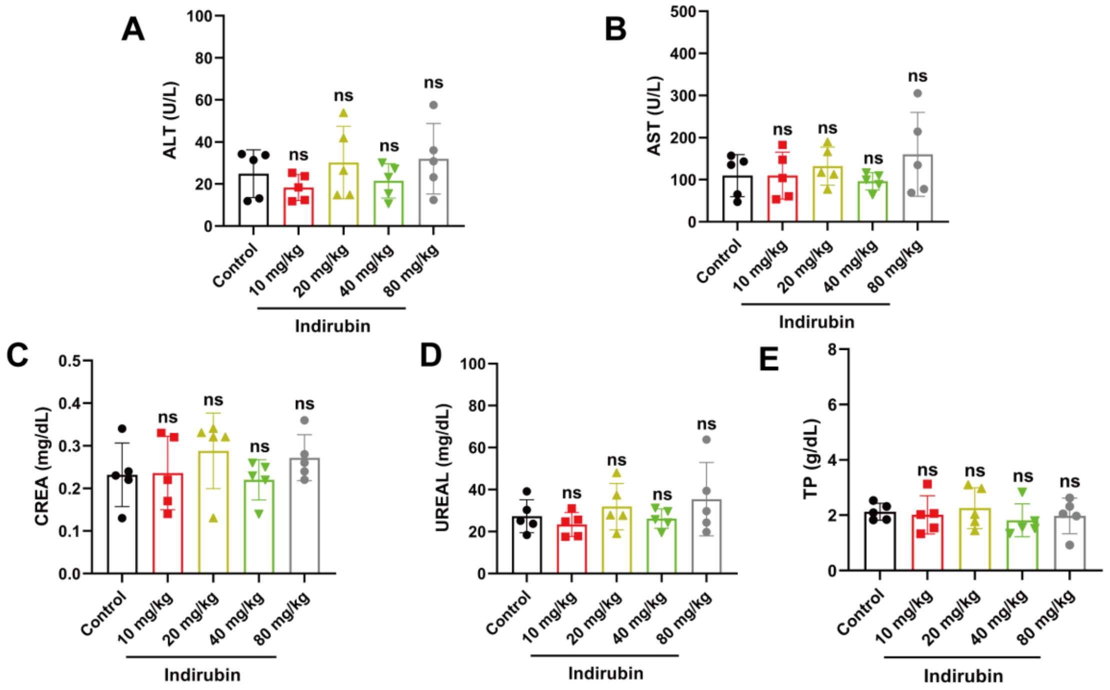

Figure S10. Serum of ALT (A), AST (B), CREA (C), UREAL (D), and TP (E) in septic mice pretreated with different concentrations of Indirubin. n=5 per group. The data are presented as mean ± SD. ns = $p > 0.05$.
